# Wearable Sensors for Remote Health Monitoring

**DOI:** 10.3390/s17010130

**Published:** 2017-01-12

**Authors:** Sumit Majumder, Tapas Mondal, M. Jamal Deen

**Affiliations:** 1 Department of Electrical and Computer Engineering, McMaster University, Hamilton, ON L8S 4L8, Canada; majums3@mcmaster.ca; 2 Department of Pediatrics, McMaster University, Hamilton, ON L8S 4L8, Canada; mondalt@mcmaster.ca

**Keywords:** wearable sensors, smart textile, remote health monitoring, body sensor network, vital sign monitoring, ambulatory monitoring

## Abstract

Life expectancy in most countries has been increasing continually over the several few decades thanks to significant improvements in medicine, public health, as well as personal and environmental hygiene. However, increased life expectancy combined with falling birth rates are expected to engender a large aging demographic in the near future that would impose significant  burdens on the socio-economic structure of these countries. Therefore, it is essential to develop cost-effective, easy-to-use systems for the sake of elderly healthcare and well-being. Remote health monitoring, based on non-invasive and wearable sensors, actuators and modern communication and information technologies offers an efficient and cost-effective solution that allows the elderly to continue to live in their comfortable home environment instead of expensive healthcare facilities. These systems will also allow healthcare personnel to monitor important physiological signs of their patients in real time, assess health conditions and provide feedback from distant facilities. In this paper, we have presented and compared several low-cost and non-invasive health and activity monitoring systems that were reported in recent years. A survey on textile-based sensors that can potentially be used in wearable systems is also presented. Finally, compatibility of several communication technologies as well as future perspectives and research challenges in remote monitoring systems will be discussed.

## 1. Introduction

Life expectancy has been increasing worldwide due to significant improvements in healthcare, and medicine, as well as due to growing consciousness about personal and environmental hygiene [1,2]. In addition, over the past several decades, there has been increasing interest in family planning [3], thereby contributing to declining birth rates around the globe. According to the World Health Organization (WHO), by 2017, the elderly population over 65 years or older are expected to outnumber the children less than 5 years of age [4]. However, this enormous aging population would create a significant impact on the socio-economic structure of society in terms of social welfare and healthcare needs. Besides this, the cost associated with health care services continues to soar because of the increasing price of prescription drugs, medical instruments, and hospital care [5]. Therefore, it is an utmost necessity to develop and implement new strategies and technologies in order to provide better health care services at an affordable price to the aging population or to the people of those areas having limited access to healthcare while ensuring maximum comfort, independence, and participation among the people.

Remote healthcare monitoring allows people to continue to stay at home rather than in expensive healthcare facilities such as hospitals or nursing homes. It thus provides an efficient and cost-effective alternative to on-site clinical monitoring [6]. Such systems equipped with non-invasive and unobtrusive wearable sensors can be viable diagnostic tools to the healthcare personnel for monitoring important physiological signs and activities of the patients in real-time, from a distant facility [6,7,8]. Therefore, it is understandable that wearable sensors play a critical role in such monitoring systems that attracted the attention of many researchers, entrepreneurs, and tech giants in recent years. A variety of application specific wearable sensors, physiological and activity monitoring systems were proposed in the literature. Apart from that, various wearable commercial products such as the biometric shirt (by Hexoskin^®^, Montreal, QC, Canada), fitness trackers (by Fitbit^®^, San Francisco, CA, USA, Jawbone^®^, San Francisco, CA, USA, Striiv^®^, Redwood city, CA, USA and Garmin^®^, Olathe, KS, USA) are now available on the market. A list of some commercial products and their principal applications is presented in Table 1. 

**Figure 1:**
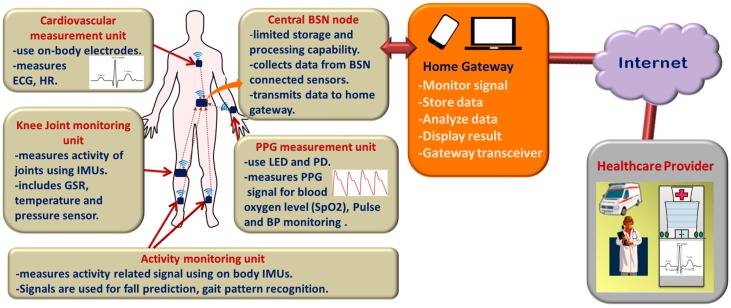
General overview of the remote health monitoring system.

Wearable devices can monitor and record real-time information about one's physiological condition and motion activities. Wearable sensor-based health monitoring systems may comprise different types of flexible sensors that can be integrated into textile fiber, clothes, and elastic bands or directly attached to the human body. The sensors are capable of measuring physiological signs such as electrocardiogram (ECG), electromyogram (EMG), heart rate (HR), body temperature, electrodermal activity (EDA), arterial oxygen saturation (SpO_2_), blood pressure (BP) and respiration rate (RR) [9,10]. In addition, micro-electro-mechanical system (MEMS) based miniature motion sensors such as accelerometers, gyroscopes, and magnetic field sensors are widely used for measuring activity related signals [6,11]. Continuous monitoring of physiological signals could help to detect and diagnose several cardiovascular, neurological and pulmonary diseases at their early onset. Also, real-time monitoring of an individual’s motion activities could be useful in fall detection, gait pattern and posture analysis, or in sleep assessment. The wearable health monitoring systems are usually equipped with a variety of electronic and MEMS sensors, actuators, wireless communication modules and signal processing units. The measurements obtained by the sensors connected in a wireless Body Sensor Network (BSN) [8,12,13,14] are transmitted to a nearby processing node using a suitable communication protocol, preferably a low-power and short-range wireless medium, for example, Bluetooth [15,16], ZigBee [15,17], ANT [15,18,19] Near Field Communications (NFC) [20,21]. The processing node, which could be a Personal Digital Assistant (PDA), smartphone, computer or a custom made processing module based on a microcontroller or a Field Programmable Gate Array (FPGA) runs advanced processing, analysis, and decision algorithms and may also store and display the results to the user. It transmits the measured data over the internet to the healthcare personnel, thus functioning as the gateway to remote healthcare facilities. The general overview of the remote health monitoring system is presented in Figure 1, although actual implemented system could differ depending on the application requirements. For example, some systems can be designed with few numbers of sensors where each of them can send data directly to the nearby gateway. In other systems, the sensors can be connected through a body sensor network (BSN) and the central BSN node gathers data from the sensors, performs limited processing before transmitting the data to the advanced processing platform. 

In order to be used for long-term monitoring purposes, wearable health monitoring systems need to satisfy certain medical and ergonomic requirements. For example, the system needs to be comfortable; the components should be flexible, small in dimensions and must be chemically inert, and nontoxic, hypo-allergenic to the human body. In addition, limitation of hardware resources is a major concern for a multi-sensor BSN system where the central node needs to handle a large amount of data coming from different sensor nodes. It also causes significant impact on the system power requirements that needs to be minimized in order to extend the battery life for long-term use. The measured and processed physiological data are, eventually, transmitted to the remote healthcare facility over the internet. Therefore, it is also necessary to use a secured communication channel in order to safeguard the privacy of sensitive personal medical data. Strong encryption techniques such as Public Key Infrastructure (PKI), Secure Sockets Layer (SSL) as well as appropriate authorization and authentication algorithms [22,23] could be implemented for enhanced data security. Finally, the system needs to be inexpensive and user-friendly in order to ensure its widespread acceptance among the people for ubiquitous health monitoring. Therefore, the critical design challenge for wearable health monitoring system is to integrate several electronic and MEMS components while ensuring measurement accuracy, efficient data processing, information security, and low-power consumption as well as user’s wearing comfort.

In this paper, we present a review on the current state of research and development in wearable systems for health monitoring by summarizing and comparing the most significant contributions in this field. Recent works on wearable sensor-based physiological parameters and activity monitoring systems are studied in Section 2, which is followed by a review on textile based flexible sensors (Section 3) that are vital for smart textile technologies. A brief discussion on wireless communication standards for the wireless monitoring system is presented in Section 4. Finally, the paper is concluded in Section 5 by illustrating some key challenges and future research directions in the field of remote health monitoring. 

## 2. Wearable Health Monitoring Systems

Non-invasive, non-intrusive sensors are indispensable elements of ambulatory and long-term health monitoring systems [6,9]. Wearable sensors, being progressively more comfortable and less obtrusive, are appropriate for monitoring an individual’s health or wellness without interrupting their daily activities. The sensors can measure several physiological signals/parameters as well as activity and movement of an individual by placing them at different locations of the body. The advancement in low-power, compact wearables (sensors, actuators, antennas, smart textiles), inexpensive computing and storage devices coupled with modern communication technologies pave the way for low-cost, unobtrusive, and long-term health monitoring system.

### 2.1. Cardiovascular Monitoring System

Electrocardiograms (ECGs) represent a non-invasive approach for measuring and recording the fluctuations of cardiac potential. This is the most widely used and effective diagnostic tool that physicians have used for decades to identify heart-related problems such as different forms of arrhythmias. 

**Figure 2:**
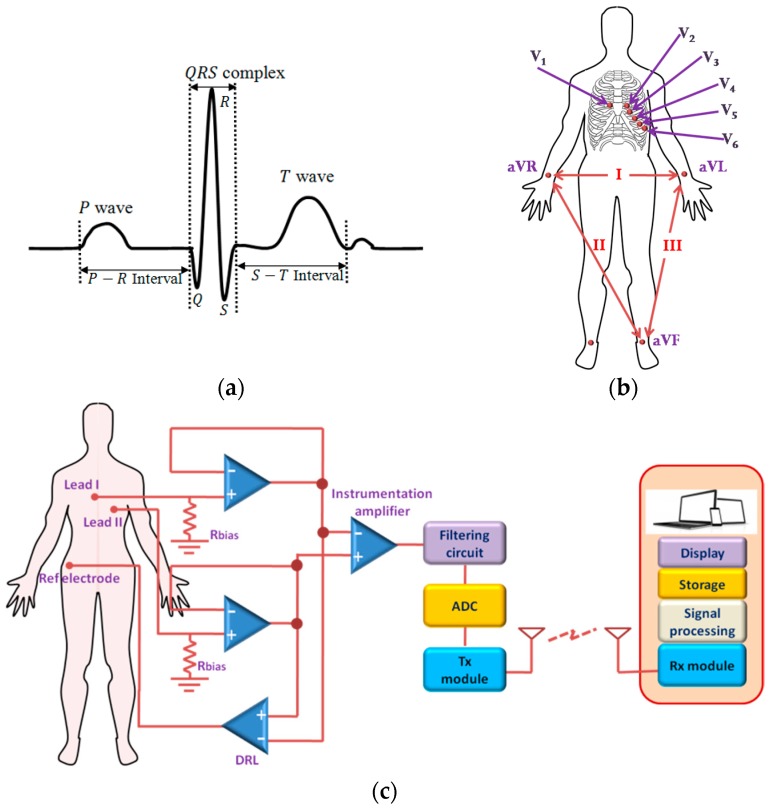
Cardiovascular monitoring: (**a**) One cycle of a typical ECG signal (not scaled); (**b**) Electrode placement in a standard 12 lead ECG system; (**c**) General architecture of ECG monitoring system.

Although many arrhythmias are not life-threatening, some results from weak or damaged heart such as myocardial infarction (MI) that may lead to cardiac arrest, if not managed immediately [24,25,26]. After a heart attack, patients are required to receive immediate medical attention, which, otherwise, may turn fatal. These complications can be avoided if any inconsistency in cardiac activity is detected and treated in an early stage that calls for outpatient ambulatory monitoring of ECG. Some rare, serious arrhythmias (e.g., Brugada Syndrome, Arrhythmogenic Right ventricular Cardiomyopathy, Long QT syndrome, hypertrophic Cardiomyopathy) are infrequent and only detected on prolonged monitoring. Figure 2a shows one cycle of a typical ECG signal. In a conventional 12 lead ECG system, electrical activities of the heart along 12 particular spatial orientations are measured using ten Ag-AgCl electrodes (hydrogel method/wet ECG), which are affixed to some specific parts of the body. Figure 2b shows the placement of the electrodes in a standard of 12-lead ECG system. The electrodes contain conducting gel in the middle of the pad that functions as a conduction medium between the skin and the electrode. This conducting gel has potential toxic and irritant effects on the skin and is thus not best suitable to use for long–term ambulatory monitoring system though currently, it is the only system available [10,27]. However, only a few numbers of electrodes are used in ambulatory ECG monitoring system at the cost of limited information (Figure 2c). A continuous ambulatory monitoring device requires a wearable and portable system that could be used comfortably without affecting an individual’s daily activities.

Andreoni et al. [28] designed a custom T-shirt and textile belts with embedded textile electrodes for monitoring ECG, HR, and R-R interval. The electrodes were made from silver based conductive yarns. Instead of using any conductive gel, the electrodes relied on body sweat, an electrolyte medium, to improve the conductivity of the skin-electrode interface and signal quality. The device also included a SpO_2_ sensor and a three-axis accelerometer for fall detection, and it could transmit the data over low-power Bluetooth 4.0. An elastic fabric-made ECG vest was presented in [29] which accommodated three electrodes, a data acquisition module and also supported robust contact of the electrodes with the skin. The electrodes were fabricated from Ni/Cu coated compressed urethane polymer foam that was enclosed by an Au-coated conductive taffeta fabric. The ECG measured by the proposed system exhibited high correlation with the simulated signal, although measurements on real subjects were not shown. Due to the conductive and flexible nature of the substrate, the skin-electrode impedance was low and remained stable over a longer period of time, thus reducing the electrode motion noise. Jeong et al. [30] proposed an ECG monitoring system where they used similar technology as [29] to develop flexible capacitive electrodes and integrated them in a chest belt. In addition to that, they used a very high bias resistor at the input of the pre-amplifier, which assisted further in reducing electrode motion artifacts. A noise cancellation and peak detection algorithm was performed on the raw ECG data to find out the QRS complex, and HR, although a detailed description of the algorithm was not provided. The authors reported achieving high sensitivity and high accuracy in detecting QRS complexes. 

The electrodes used in the ECG systems presented in [28,29,30] were in direct contact with the skin. Nemati et al. [10] embedded a small, low–power, wireless ECG monitoring system in a stretchable belt where three capacitive electrodes were integrated into a cotton T-shirt, thus enabling ECG measurements to be performed over the cloth. The cotton functioned as the dielectric material between the electrode and the skin. The signal processing and communication modules were mounted on a small two-layer PCB board. Power consumption was minimized by selecting low power electronic components for the system, ANT protocol for wireless communication as well as by adopting idle mode signal sampling technique. However, the rigid electrodes can be inconvenient to the users and may induce motion artifacts in the signal. 

In order to minimize the common mode interference, an additional driven right-leg (DRL) electrode was used in [10,29,30]. This DRL electrode is usually placed at a distant site, far from the measurement electrodes and thus requires a long wired connection, which may not be convenient for wearable and long-term monitoring systems. Komensky et al. [31] proposed an ECG monitoring system without the DRL circuit, where only two active capacitive sensors were embedded in an elastic chest band. In order to increase and stabilize the input impedance, two anti-parallel connected diodes were used for biasing that has advantages over resistors [30] of low thermal noise and fast recovery time. The ECG measurements on stable subjects were reasonably well, although the P waves were indistinguishable, which might be attributed to the electrodes’ position on the body or the absence of the common electrode. On the other hand, the measurements during walking were greatly affected by the motion artifacts, but the QRS complexes were still recognizable.

Many researchers have developed and made use of piezoelectric pressure sensors for measuring the HR by sensing the arterial pulse wave generated by the periodic contraction and relaxation of the heart. A wireless HR monitoring device was presented in [32] that could estimate HR from the pressure variation in the ear’s canal surface. A piezoelectric film pressure was used to sense the in-ear pulse waves (EPW) and convert it to an electric current. A knowledge-based algorithm was implemented in a microcontroller that could detect the pulse peak in real time from the signal after performing a morphological conversion. However, the pressure variance, and thereby the peak height of the pressure waves can be affected by body movements that introduce error in HR estimation. In addition, an ear-mounted device is inconvenient for long-term use. A similar system was proposed in [33] where the authors developed a polymer-based flexible piezoresistive pressure sensor that can sense pressure variation on the skin caused by the pulsation of arterial blood. They used carbon black/silicone rubber nanocomposite as the flexible piezoresistive material. High sensitivity and linearity of the pressure sensor was achieved by forming microstructures at the contact surface of two piezoresistive layers. They also proposed a low-cost analog signal processing (ASP) system that could perform denoising, data processing, and HR measurement.

Yoon et al. [34] designed a skin attachable piezoelectric pressure sensor and demonstrated its usability in HR estimation by sensing the pulse wave in human artery. The pressure sensor was fabricated on a polyimide substrate with a small window. A thermally evaporated silver electrode was spin-coated with a polyvinylidene fluoride-trifluoroethylene (P(VDF-TrFE)) piezoelectric layer. The pressure variation in the radial artery causes mechanical stress on the piezoelectric layer, resulting in potential variation across the electrodes. Tajitsu et al. [35] embedded a piezoresistive pressure sensor in a wristband for HR monitoring. The piezoresistive material was made from nonwoven acrylate-modified polytetrafluoroethylene (PTFE) fabric that was fabricated using electro-spinning. The PTFE was deposited on an aluminum electrode on a polyethyleneterephthalate (PET) film. The pulse wave measured from the wrist by this sensor had similar pattern as the ECG signal and showed high accuracy as well as less vulnerability to motion-induced noise.

Some researchers have exploited system-on-chip (SOC) technologies to integrate both analog and digital signal processing units for on-chip ECG signal processing. Izumi et al. [36] developed a wearable system that incorporated a near field communication (NFC) module, a three-axis accelerometer, and an ECG processor chip. The chip was designed to perform data acquisition, process ECG and accelerometer signals, and communicate with the smartphone. The R-peak detection and HR estimation was performed by utilizing short-term autocorrelation (STAC) between a template signal and the measured signal. The chip was fabricated using a standard 130-nm CMOS technology. The system was reported to consume ~13.7 µA current and perform monitoring for about 24 days using a 35 mAh battery. An ultra-low-power ASIC was designed for cardiovascular monitoring in [37], which was fabricated using a standard 0.18 µm CMOS technology and encompassed a two-stage Miller-compensated programmable gain amplifier (PGA), QRS and baseline amplifiers, DC voltage generator and a comparator. The PGA offered wide dynamic range, self-biasing capability, and low supply voltage requirement. The ‘QRS Amp’ and a ‘Baseline Amp’ filtered the signal and isolated QRS signal from the baseline drift. A DC voltage was added to the baseline drift and R–peaks were detected by comparing the QRS complex signal with the shifted baseline. The system required only 58 nW of power and can operate continuously for one year with a 0.7 mAh thin-film battery, thereby making it suitable for long-term monitoring applications. Helleputte et al. [38] proposed a design of a 3-channel bio-potential acquisition integrated circuit. Each channel measures ECG as well as electrode-tissue impedance (ETI), which is found to be strongly correlated with the motion artifacts. Motion artifacts are estimated in real-time using an adaptive LMS filter and subtracted from the ECG signal before amplification.

The system was reported to consume low power and suppress slow varying (<10 Hz) motion artifacts in ECG. Table 2 presents a comparison among the cardiovascular monitoring systems discussed above.

### 2.2. Activity Monitoring System

**Figure 3:**
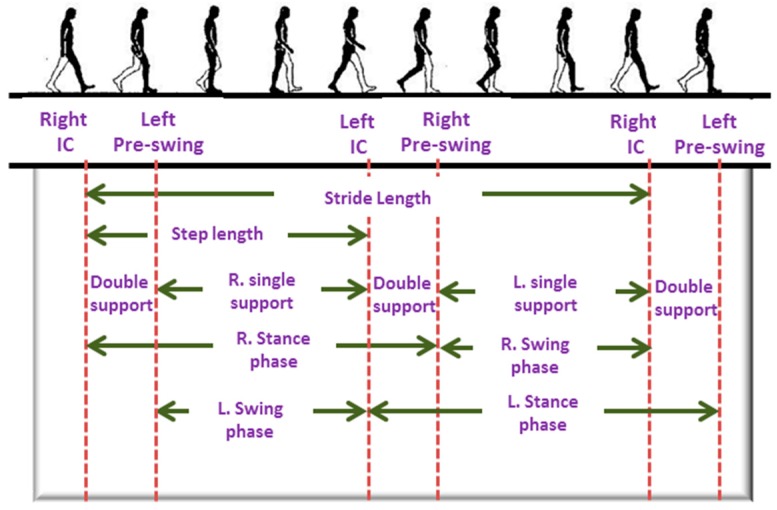
A typical human gait cycle.

Monitoring an individual’s physical activities and locomotion can be useful in rehabilitation, sports, early detection of musculoskeletal or cognitive diseases, fall and balance assessment. It has been reported that an individual’s walking patterns are strongly associated with their health condition [39]. Walking involves several joints including spine, hip, knee, ankle, tarsal and metatarsal joints. It equally involves several muscles, for example, muscles of back, around hip joints, thigh, calf muscles and several small muscles of foot. A typical walking cycle is shown in Figure 3. Walking, especially turning event requires good balance and coordination among different parts of the body, which is controlled by the cerebellum. Therefore, any abnormality in walking patterns can be indicative of possible musculoskeletal, central nervous system (CNS) or peripheral nervous system diseases. 

The walking patterns of ailing people tend to differ from that of normal healthy people. For example, people at the early onset of neurodegenerative disorders such as Alzheimer’s or Parkinson’s tend to exhibit different walking patterns [40,41]. One of the possible early signs of Parkinson’s disease is small and shuffled walking steps. Besides that, a person at the early stage of Parkinson’s may experience difficulties in starting, stopping and turning events while walking. They may show loss of associated movements. On the other hand, elderly people, owing to their declining motor control and muscle strength are usually more vulnerable to fall, which, if occurs, may cause joint injuries, hip and bone fractures and traumatic brain injury. These injuries demand longer recovery time, restrict physical movement and affect the daily activities of the individual. Indeed there is a strong correlation with mortality and fall-related fractures [41]. Quantitative analysis and assessment of the gait can be useful for early detection of several diseases, fall prediction as well as during the rehabilitation period after an injury.

**Figure 4:**
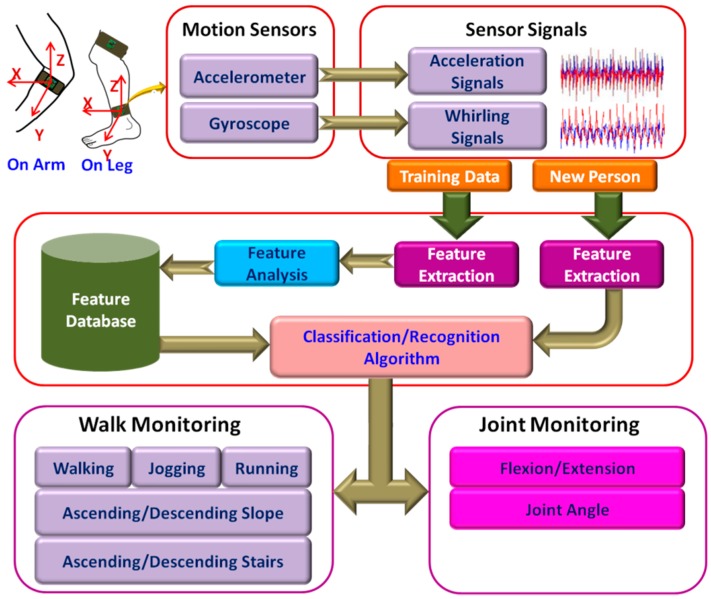
Schematic representation of activity monitoring systems.

Home-based fixed position monitoring, for example, camera-based systems are useful tool for activity monitoring [42,43]. These systems are capable of recognizing complex gait activities. However, such systems restrict the movement of the user within a specific range. Apart from that, these systems are complex and expensive. In recent years, use of wearable motion sensors such as accelerometers, gyroscopes, and magnetometers are gaining in popularity for measuring human gait activities [6,11] in real time. The sensors measure linear and angular motion of the body from which a number of key features are extracted. Table 3 presents a list of key features that can be extracted from the signals. These features are used to quantify and classify human gait events. A schematic of the activity monitoring system based on accelerometers and gyroscopes is presented in Figure 4. 

Derawi et al. [44] implemented an activity and gait recognition system on a smartphone. They measured the gait data with the help of the accelerometer in the smartphone. An application software was developed for the smartphone that performed detection, normalization, averaging of gate cycles, activity, and gait recognition. The algorithm used Manhattan distance metric to compare the average gait cycle of a test sample to three different template gait cycles that correspond to three different walking speeds. The authors exploited both statistical and machine learning approaches in order to classify among three different walking speeds and achieved high accuracy from the support vector machine (SVM) approach. However, these methods rely on local peak and valley detection, which is sensitive to variations in walking speed and/or style. Debraj et al. [45] proposed an activity recognition system that used multi-modal sensors to detect complex daily activities. Each measurement unit, which was a smartphone in this case, comprised an accelerometer and a gyroscope for activity measurement; temperature, humidity, and barometric pressure sensors for environment sensing; and Bluetooth beacon for location assessment. Four such measurement units were attached to waist, back, leg, and wrist. After extracting a set of suitable features from the preprocessed sensor data, classification was performed separately on each unit using a modified conditional random field (CRF) algorithm. Final recognition was performed by assessing the classifier decisions from each unit based on their relevance to the body positions. The authors reported on classifying 19 in-home activities including using refrigerator, cleaning utensils, cooking, sitting and eating, using the bathroom sink along with normal daily activities with high accuracy. Further study and development are required in order to realize it in a fully wearable system and implement it for remote monitoring of the elderly. In addition, more investigations on machine learning classifiers are necessary to achieve a more robust recognition algorithm. 

Bertolotti et al. [46] designed a lightweight, wireless wearable device for assessing the balance control abilities of the body by measuring the limb movements for a longer period of time using an accelerometer, a magnetometer, and a gyroscope. Several units can also be connected in a body sensor network (BSN) for achieving more detailed measurements. The reliability of the system was validated by comparing the center-of-mass (CoM) displacement estimated from the measurement with that obtained from a Wii Balance Board (WBB). Further research is necessary in order to extract a suitable set of features from the measurement data and classify subjects in terms of fall vulnerability. Panahandeh et al. [47] proposed a human activity and joint classification and gait analysis algorithm based on continuous hidden Markov model (HMM). They devised a chest mounted activity measurement system that performed inertial measurements using one tri-axial accelerometer and gyroscope. They proposed a feature extraction method, which is based on calculating discrete Fourier transform (DFT) coefficients from segments of the signal. The authors achieved high classification accuracy among different walking events. Due to the absence of wireless connectivity, the subjects had to carry the laptop during the experiment that could affect the normal human locomotive behavior. Besides this, the feature extraction was performed in an ad-hoc basis. The system can be further improved by incorporating wireless connectivity and developing better feature extraction algorithms.

Chia Bejarano et al. [48] proposed an adaptive algorithm for real-time gait-event detection. They attached both inertial motion and magnetic sensors on the shanks and measured the angular velocity and flexion-extension angle for each leg. After initial calibration, the algorithm employed a threshold-based state machine in order to detect three gait-events: initial contact, end contact and mid-swing for each leg at different walking speeds. This algorithm exhibited high detection accuracy as well as low detection delay, thus making it suitable for real-time applications. However, further validation with a larger number of subjects is necessary. In addition, the measurement from the magnetometer that was used by the Kalman filter can be affected in the presence of ferrous materials which can cause false detection. Another inertial measurement unit (IMU)-based gait recognition algorithm was presented in [49] where three sets of IMUs were placed on the center, left, and right of the back using a waist belt. The step signals were first segmented from the likelihood of heel strike computed using a scale-space technique. An algorithm based on inter-class relationships extracted the feature vectors. It also performed tilt correction using the gyroscope on the IMUs and employed an iterative matching algorithm to resolve the inconsistency associated with the sensor orientation. The researchers carried out experiments on a large number of subjects and reported an average classification accuracy of 93%. 

An intensity-independent activity recognition method was proposed in [50] that utilized the uncertainty among the clusters. They selected a fixed set of features from the accelerometer data during the training phase and clustered the data into group of activities by employing K-means or the Gaussian mixture model (GMM) algorithms. The mean and variance of each activity cluster were used to form a stochastic activity model (SAM) matrix. In the recognition phase, the appropriate group for the test sample was determined by employing the nearest-neighbor algorithm on the SAM matrix. Test results showed that the system could discriminate walking and running across three different intensity levels with a high degree of accuracy. Ghasemzadeh et al. [51] proposed a real-time feature selection algorithm for wearable systems, which was designed to minimize energy requirements during classification. This algorithm identified and discarded irrelevant and redundant features, thus increasing the learning speed and optimizing the power requirement for the system. The redundancy analysis was performed by using symmetric uncertainty among the features producing strongly correlated features. A graph model was deduced from the correlation analysis, which was used by an integer programming, and a greedy approximation based optimization algorithm to find out the optimal features. They used six sets of IMUs for movement monitoring and reported to obtain 30% of energy savings while achieving 96.7% classification accuracy among 14 sets of movements. Further development of algorithms for dynamic feature selection as well as sensor unit activation may result in an improved power-optimized system. 

A walking-phase dependent parameter optimization algorithm has been presented in [52] for efficient classification of locomotion modes. The walking phase was detected from the signals of the pressure sensors embedded in insoles. The gait signal measured by two IMUs and pressure insoles were segmented into four walking phases. The authors, then optimized the feature vector, classification algorithm, and window size separately for each phase. This algorithm exhibited high recognition accuracy (~96.5%) and fast computation time when the performance of the complete gait cycles is considered for optimization as compared to the conventional methods of evaluating each phase of a gait cycle separately. Cristiani et al. [53] devised an electronic insole for long-term monitoring of motor activities. The insole housed a tri-axis accelerometer, humidity and temperature sensors, and four pressure sensors. The sensors were connected to a microcontroller having an integrated 2.4 GHz transceiver in it. The onboard flash memory allows storing up to 10 h of measurement data. The insole was comfortable to use in regular motor activities and thus can be used to monitor a subject’s movement for longer periods of time. However, the insole requires initial calibration for each individual in order to make an inclination correction. Integrating a gyroscope in the insole for inclination measurement might be useful to avoid this initial calibration phase. Another shoe-based activity monitoring system was presented in [54] where recognition is performed by rejecting unreliable data while employing the classifiers. A set of nine features were calculated from each measurement obtained from each of five insole pressure sensors as well as from the accelerometer, which was attached to the heel of the shoe. The authors tested both SVM and multilayer perception (MLP) classifier and rejected those data points residing near the cluster boundaries. Very high recognition accuracy (99.8% ± 0.1%) was achieved by applying MLP on raw measurement data. However, the tests were performed only on nine subjects. Besides this, the rejection threshold was determined in a heuristic manner, whereas an adaptive way of calculating the threshold could be more useful for practical applications. 

Friedman et al. [55] designed a wearable wrist and finger joint monitoring system using magnetic sensing technology. The system comprised of a neodymium ring, a sensing and data storage unit. The ring worn on the index finger generated a magnetic field, which was measured by two tri-axial magnetometers mounted on the wrist-worn sensing unit. A radial basis function network estimated the angles of the wrist and finger joints from the measurements. The authors reported a highly accurate estimation of angular distance for wrist joints. However, with a high standard deviation, finger flexion-extension estimation was poor. This estimation can be improved by ensuring accurate initial calibration for each user. An approach for human joint angle estimation by combining kinematic arm models with the state space algorithm has been presented in [56]. Inertial measurements were obtained from three sets of IMUs attached on the upper arm, forearm, and, wrist of a robot arm. The state model incorporated random drift models and zero-velocity updates that reduced the effect of sensor drifts. The model also considered the physical constraints of joint movements in order to achieve higher estimation accuracy. The authors estimated joint angles using the Unscented Kalman filter (UKF) [57], an improved algorithm over the Extended Kalman filter (EKF) especially developed for systems with higher nonlinearities. They achieved a high degree of estimation accuracy at different intensities of arm motion. Nevertheless, validation of this algorithm on human subjects is necessary. It should be noted that Kalman-based solutions are computationally intensive and also need a high sampling rate in human activity monitoring applications. Therefore, realizing the systems for real-time applications can be challenging.

Hsu et al. [58] implemented an algorithm for gait and balance analysis in an IMU based wearable system. For gait measurements, two IMUs were attached to the top of each shoe, and one IMU was attached to the back of the waist for balance analysis. After the detection of strides, the gait cycles were further decomposed by using the toe-off and heel-strike points obtained from the pitch signals of the gyroscope. A number of gait parameters corresponding to walking speed, rhythm, and, variation were calculated from the decomposed signal. The balance was measured by calculating the sway speed from the center of mass (COM) analysis. A wearable fall detection system was proposed in [59] that could determine fall events by employing acceleration and orientation thresholds. The acceleration thresholds were obtained at the training phase from SVM, and the postural orientation thresholds were determined from the subject’s tilt angle. The system used Madgwick’s orientation filter for reducing magnetic distortion and gyroscope drift, resulting in high estimation accuracy. The IMU was placed on the waist and could communicate over Bluetooth. The system analyzed the RMS data obtained from the accelerometer and the orientation filter and could detect fall events using a threshold based algorithm. This allows implementing the algorithm for real-time applications in a low profile microprocessor. The algorithm was reported to achieve a high degree of accuracy and sensitivity. Table 4 presents a comparison of the key features and performance characteristics among the activity monitoring systems discussed above. 

### 2.3. Body Temperature Monitoring System

Body temperature is one of the vital signs that can reflect health conditions. Body temperature increases in infections, malignancy and many inflammatory conditions. Only serial temperature measurements over a long period of time rather than spot checks may prompt the diagnosis. It was reported that core body temperature (CBT) has strong influence on different physiologic conditions as well. Disruptions in the body temperature rhythm are reported to be associated with different types of insomnia [60]. For example, patients suffering from delayed sleep phase insomnia have ~2 h of delay in reaching their minimum CBT compared to the group of good sleepers [60]. Besides this, variation in body temperature rhythm with menstruation cycle was also observed in some studies [61,62]. Researchers [63] also reported observing a correlation between body temperature and initial stroke severity, infarct size, mortality among stroke patients. It was observed that the infarct size worsens by ~15 mm with 1 °C increase in body temperature. In addition, a strong correlation between body temperature and cognitive functions was also reported in the literature [64,65]. 

Various noninvasive approaches for continuous body temperature monitoring were reported in the literature. Buller et al. [66] proposed a Kalman filter-based model that is inspired from the work reported in [67] where the authors estimated core body temperature from the heart rate. Although the model is validated with only a few subjects and accumulates error with time, it has the potential to be embedded in wearable ECG monitoring systems. Further study and development are also necessary in order to validate the estimation accuracy in the presence of rapid physical movement of the subject, for example, running, exercising etc. A noninvasive, dual-channel body temperature measurement system was reported in [68] that can measure temperature with an accuracy of ±0.1 °C within the range of 16–42 °C and also transmit data over a Bluetooth communication platform. The device has two temperature probes, each containing a digital temperature sensor. The probes measure temperature from two ear canals simultaneously from which the mean temperature is calculated. However, because of the ear-canal probes, the system may not be feasible for long-term monitoring. 

Boano et al. [69] demonstrated an unobtrusive, wireless body temperature monitoring system that can be worn by an individual for a long period of time. Two sensor units were attached to the skin that can measure and send data to a more powerful body-worn central unit, thus forming a star-type body sensor network (BSN). The central unit sends the data to a computer that can communicate with health care facilities over the internet. The authors achieved an accuracy of 0.02 °C over the temperature range of 16–42 °C. They also demonstrated the detection of circadian rhythm using this system. The authors later developed a wireless core body temperature monitoring system for marathon runners that measures the tympanic temperature [70]. A non-invasive, wearable temperature monitoring systems was designed for neonates in [71]. They integrated a negative temperature coefficient (NTC) resistor in a belt made of soft bamboo fabric. Instead of hard wires, the authors used soft and flexible silver plated nylon yarns as the conductive medium woven in the belt. The system exhibits an accuracy of 0.1 °C when compared with the measurement obtained from a standard thermometer. A reliable and stable connection between fabric wires and the sensor could is critical and was not investigated in this research. However, the designed system can be integrated in a smart jacket or smart belt. Thus, it has a great potential to be used in non-invasive, long-term monitoring, even for adults. Mansor et al. [72] demonstrated the implementation of a wireless body temperature monitoring system using comercial sensors. The temperature sensor, which comes with an integrated ZigBee wireless node, measures and transmits data to a microcontroller. The microcontroller sends data to a remote server over a wireless local area network (WLAN). The authors used Arduino Ethernet shield for developing the prototype. Similar temperature and heart rate monitoring systems were reported in [73,74]. 

Some researchers exploited RFID technology for body temperature monitoring systems. Vaz et al. [75] designed and fabricated a low power RFID temperature sensor chip in 0.35-μm CMOS process. The chip along with a matched impedance dipole antenna measures the temperature with an accuracy of ~±0.1 °C within the typical range of human body temperature and can communicate with a 2 W ERP (effective radiated power) output reader at a frequency of 868 MHz from a distance as far as 2 m. A RFID based real-time, continuous body temperature measurement system that is fabricated on a poly ε-caprolactone (PCL) membrane was developed by Milici et al. [76]. The system comprises a wearable RFID tag operating in the ultra-high frequency (UHF) band and a RFID microchip, EM4325 that has the capability of measuring temperature with a resolution of 0.25 °C as well as functioning as a regular RFID transceiver. The measured temperature was read by a short range RFID reader, connected with a linear-polarized antenna. Although the linear-polarized antenna offers longer read range, they are very sensitive to tag orientation. A circular-polarized read antenna could circumvent this problem. 

In order to measure the core body temperature, Sim et al. [77] designed a system by embedding a dual-heat-flux probe and two double-sensor thermometers [78] into a neck pillow. Since the jugular vein passes by the neck, the skin temperature over it would have a strong correlation with the core body temperature (CBT). The temperature estimated by this system was found to be similar with that measured by an IR thermometer from the tympanic membrane. The temperature curves obtained from three sensors are distinct and also vary with different sleeping positions which may provide sleep-related information. The authors also proposed a curve-fitting method in order to improve the inherent slow response time of the dual-heat-flux thermometer. Although the system is designed for patients to use in beds, embedding the system in the collar of the shirt or textile band is also possible. Kitamura et al. [79] developed a temperature sensor probe that can measure CBT from the surface of the skin. The circular metal probe comprises two heat-flow channels for two different thermal resistances and each channel has a pair of temperature transducers attached at both ends. The presented system demonstrates a long initial response time but higher accuracy (97% correlation with the measurement from zero-heat-flow thermometer) once it reaches the equilibrium temperature. The response time can be improved by using an Al probe instead of the Cu probe. Table 5 presents the comparison among the body temperature monitoring systems discussed above. 

### 2.4. Galvanic Skin Response (GSR) Monitoring System

The autonomic nervous system (ANS) controls and regulates the response of the body to internal or external stimuli by balancing the activities within its two subdivisions: sympathetic and parasympathetic nervous systems [80]. The parasympathetic system, which is also termed as “rest and digest" system conserves and restores energy of the body. On the other hand, the sympathetic system triggers what is often referred to as fight or flight response by increasing metabolic output to deal with the external stimuli. Increased activity of the sympathetic system accelerates heart rate, increases blood pressure, and sweat secretion, as well as prepares the body for motor action by pumping more blood to muscles, lungs, and brain. 

**Figure 5:**
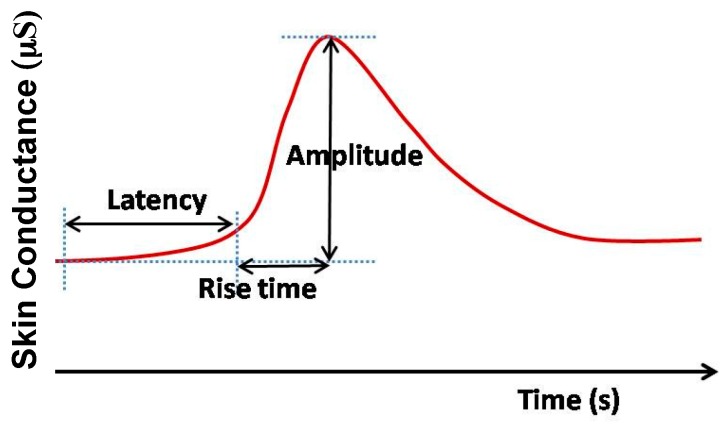
Typical galvanic skin response (GSR) signal (not to scale).

Although currently, this information is of no significant clinical use, there is growing interest in many conditions including dysautonomia, Postural orthostatic tachycardia syndrome (POTS), and Inappropriate tachycardia syndrome. Increased sweat secretion from eccrine glands fills the sweat ducts. Sweat, being a weak electrolyte, increases the conductance of the skin with increased secretion. Therefore, variation in skin conductance (Figure 5), which is also referred to as electro-dermal activity (EDA) or galvanic skin response (GSR), reflects the activity of the sympathetic nervous system and provides a simple, sensitive and reliable parameter for assessing the sympathetic nervous activities associated with stress and emotion [81,82].

**Figure 6:**
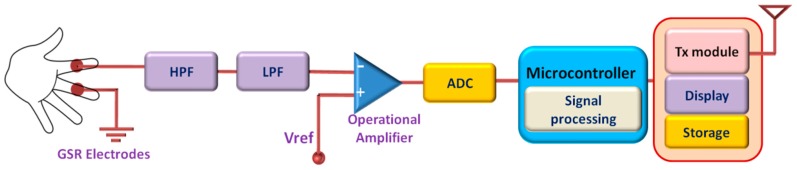
Schematic diagram of the GSR monitoring system.

Usually, GSR is measured from the part of the skin having a large number of sweat glands such as the palm, fingers, or soles of the feet. In active measurement, a DC voltage is applied across two on-body electrodes and the skin conductance is obtained from Ohm’s law by measuring the current. The earlier researches in this field mostly focused on time-limited GSR measurement systems used in laboratories and health care facilities. The development of low-power and wearable technologies opens up a new window for unobtrusive GSR monitoring that can be worn for a longer period of time [83]. Long-term monitoring of GSR allows to observe and assess the response of the sympathetic nervous system for a longer period of time and can potentially unfold important physiological information that cannot be obtained by limited time monitoring. Besides this, wearable GSR monitoring system allows patients to monitor GSR level in their home environment to provide a better assessment of their psychophysiological condition than the evaluation made at laboratories or hospitals from short-term measurement [83,84]. A schematic diagram of a wearable GSR monitoring system is presented in Figure 6.

A low-cost GSR sensor has been presented in [85] that was embedded in a wristband. The sensor module is small in size (20 mm × 30 mm × 0.8 mm) and accommodates measurement, processing, and communication functionalities. The wrist-worn sensor measures GSR from the dorsal forearm and transmits the data with a Gaussian Frequency Shift Keying (GFSK) transceiver. The elastic nature of the wristband provides stable, consistent and undisturbed electrode-skin interface, thus minimizing motion artifacts in the GSR measurements. Sugathan et al. [86] integrated a set of non-invasive sensors in a shirt that facilitates real-time measurement of HR, GSR, and body temperature. They used an Arduino-based wearable computing device (LilyPad) as the primary computing platform. Integration of storage and wireless communication modules could make the system more feasible for long-term and remote health monitoring applications.

A wearable GSR sensor has been reported in [87] that can conduct measurement from the back of the body and transmit data over the Bluetooth platform. The sensor was fabricated on a flexible PCB, which was covered with silicon providing stable contact with the curved body surface. Here [87] a dry conductive polymer foam was used as the sensing material for the flexible electrodes. The flexible nature of the electrodes offers stable and reliable skin-electrode interface as well as wearing comfort to the user. The GSR measured by the reported system had good correlation (average ~0.768) with the reference GSR system, although the validation was performed on a very small number of subjects. Garbarino et al. [88] developed a multi-sensor wristband (Empatica E3) that included GSR, PPG, temperature, and motion sensors. The GSR sensor offered high dynamic range measurement between 0.01 µS to 100 µS at 900 pS resolution. The data acquisition device has a dimension of 4 cm × 4 cm and was embedded in a wristband. It was capable of measuring and logging data from all the four sensors continuously for 38 h. The system could also stream data in real time using Bluetooth LE wireless medium. The long battery life, low power wireless connectivity, and multi-parameter monitoring capability are key features of the proposed system. 

Guo et al. [89] presented a GSR monitoring system and developed an algorithm to identify human emotions. A 20-dimensional feature vector was extracted from the pre-processed GSR signal. The authors then employed Sequential Floating Forward Selection (SFFS) algorithm on the feature vector for classifying the data into four sets of emotions: amusement, fear, sadness, and relaxation. However, the method was validated only on a few subjects. An investigation on GSR data was performed by Setz et al. [90] in an attempt to distinguish cognitive load and stress. The authors separately measured GSR from the fingers of a number of subjects working in two artificially created psychological conditions mimicking cognitive load and stress. A wearable monitoring system was used that measured and transmitted GSR signal via a Bluetooth wireless link. It was observed that among 16 selected features of GSR signal distributions, the GSR peak height and the instantaneous peak rate provided better result in differentiating cognitive load from stress. Crifaci et al. [91] studied the feasibility of wearable, wireless HR and GSR monitoring system in assessing the stress level and the function of an autonomic system in ambulatory condition. ECG and GSR measurements were taken from two groups of young adolescents. The authors performed statistical analysis on the features extracted from the ECG and GSR. The group of healthy people exhibited significantly higher variance and standard deviation in their GSR level compared to the group of people affected by the eating disorder anorexia nervosa. Subramanya et al. [92] performed an investigation on the correlation between blood pressure (BP) indexes and GSR exists. The authors implemented a wearable GSR monitoring system based on the design presented in [93] and attempted to correlate the measured GSR data with four BP indexes: systolic BP (SBP), diastolic BP (DBP), mean arterial pressure (MAP), and pulse pressure (PP). The study showed that among the four indexes PP is highly correlated with GSR. Research [94] showed that PP is a vital indicator of cardiovascular risks and a 10 mm Hg increase in PP causes approximately 20% increase in cardiovascular death. Therefore, the correlation observed in [92] between PP and GSR could be significant for cardiovascular assessment. Table 6 presents the comparison among the GSR monitoring systems discussed here.

### 2.5. Blood Oxygen Saturation (SpO_2_) Monitoring Systems

Peripheral capillary oxygen saturation (SpO_2_) is a measure of the amount of oxygenated hemoglobin in the blood. The oxygen level in blood can be decreased due to health conditions such as cardiovascular diseases, pulmonary diseases, anemia and sleep apnea. It can also be reduced following excessive physical activities. It is essential to maintain an adequate amount of oxygen (>94%) in the blood to ensure proper functioning of cells and tissues [95,96]. Therefore, it is important to monitor SpO_2_ continuously, especially for persons having respiratory and heart-related diseases.

**Figure 7:**
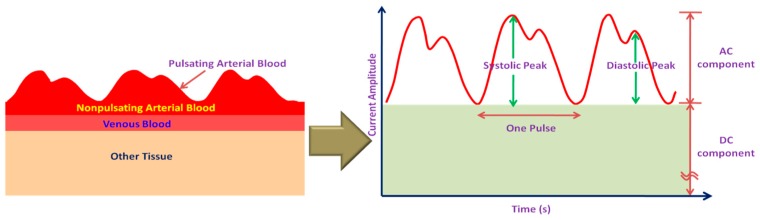
Arterial blood flow and corresponding PPG signal (not scaled).

Pulse oximeters are widely used as a fast, non-invasive mean to measure the oxygen level in blood. The estimate of SpO_2_ is from the absorption characteristics of blood in response to red (660 nm) and infrared (940 nm) light. When hemoglobin becomes oxygenated, its color changes from dark red to bright red that reduces the absorption of red light. The light absorption in blood also varies with the change of arterial blood volume during systolic and diastolic phase of the heart, resulting in a time varying signal called as photo-plethysmograph (PPG). The schematic representation of arterial blood flow and its corresponding PPG signal is shown in Figure 7.

The intensity of the transmitted light (I) can be determined by the well known Beer-Lambert law that states that the intensity of transmitted light decreases logarithmically with the concentration of oxygenated $\left(C_{O}\right)$ and deoxygenated $\left(C_{d}\right)$ hemoglobin, the absorption coefficients of both $\left(\alpha_{O},\alpha_{d}\right)$ at a particular wavelength, and on the thickness (l) of the arteries according to:

(1) $$I=I_{0}exp\left(-\left(\alpha_{O}C_{O}+\alpha_{d}C_{d}\right)l\right)$$

The SpO_2_ is calibrated from the PPG signal by measuring and comparing the intensity of transmitted light at two wavelengths. The PPG signal can also be used to determine the respiratory rate, pulse and heart rate [97,98]. It can also be used along with the ECG signal for estimating BP from the pulse transit time [99,100]. 

**Figure 8:**
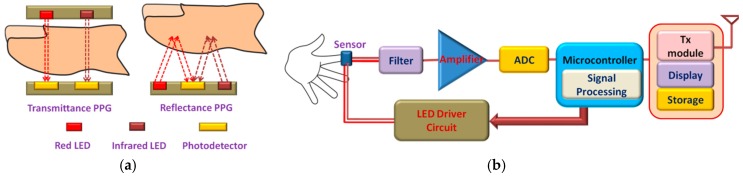
Photoplethysmography (PPG): (**a**) Different approaches for measuring PPG; (**b**) Schematic diagram of the SpO_2_ monitoring system.

The pulse oximeter usually utilizes red and infrared light emitting diodes (LED) as the light sources. The residue lights after absorption are detected by the photodetector (PD). PPG or SpO_2_ sensors can be classified into two categories based on the working principles: transmittance and reflectance oximetry (Figure 8a). In transmittance oximetry, the LEDs and PD are placed on opposite sides of a transparent section of the body such as an earlobe, a fingertip or on the palms or soles of small babies. Light transmitted through this section is collected by the PD. Currently, fingertip based transmittance pulse oximeters are widely used for PPG signal measurements [101]. However, this method is inconvenient for long-term monitoring. In reflectance oximetry, the LEDs and PD are placed side by side on the same body surface and intensity of the reflected light is measured by the PD. It offers flexibility for measuring PPG signal from different locations on the body that makes it more suitable for the non-invasive wearable platform. The schematic diagram of the SpO_2_ monitoring system has been shown in Figure 8b.

A transmittance SpO_2_ sensor probe has been proposed in [102] that could be embedded in a finger ring, unlike the conventional fingertip probes. A novel distribution of optical sensors and LEDs around the phalanx and mounted them on a flexible PCB was proposed. Experiments were carried out on 10 subjects and the results were in good agreement with that measured by commercial finger-tip oximeters. This ring-type SpO_2_ sensor probe could be useful for monitoring arterial oxygen level and heart rate for a long period of time. Guo et al. [100] integrated a vital sign monitoring system in a chest band, embedded with micro-machined Pt electrodes. The band contains a miniaturized PCB that comprises an ECG analog front-end, a driver circuit for an ear-worn PPG probe and UART/wireless transceiver. The system measures ECG, and PPG signals at a rate of 200 Hz and transmits the data using BSN (body sensor network) node to a hand-held device (e.g., PDA) where motion induced noise are removed by using a wavelet de-noising process. The PDA calculates and displays heart rate, BP and SpO_2_ and can transmit the data through GSM to healthcare personnel, if necessary. 

A reflectance probe has been designed by Cai et al. [103] for measuring PPG signal that can be worn as a wristband. The wristband is also equipped with an RF transmitting module that facilitates wireless communication between the measurement system and the health care center. Experimental results show that the system can detect the change in oxygen level in blood effectively and thus could be useful in non-invasive, continuous and remote monitoring systems. Chen et al. [98] demonstrated a non-invasive oxygen saturation monitoring system for newborn babies. The reflectance sensor is embedded in soft fabrics that makes it suitable for a wearable, long-term monitoring system. The system measures HR, SpO_2_ and can transmit the data by an RF transceiver. Experimental result showed that the measured data closely follows the measurement obtained from the commercial monitoring system. However, further improvement in the design is needed in order to minimize the impact of motion artifacts that causes false reading. A small, low-cost wearable reflectance pulse oximeter was proposed in [99] that can provide quality PPG signal without using any filter circuit. The PPG signal is sampled at a rate of 240 Hz which is then transmitted to a host computer by a ZigBee transceiver module or by a mini-USB. Further processing is done in computer in order to remove the ambient noise and slowly varying motion artifacts. This system can be used to monitor HR, respiration rate, SpO_2_, and BP. 

Huang et al. [104] performed the Monte-Carlo simulation of optical interaction with human tissue in order to investigate the feasibility of a ring-type pulse oximeter with multiple detectors [105,106]. The optimum positions of the light source and the detectors are determined from the simulation results. It has also been reported that multi-detector sensor improves the stability and the light gathering efficiency. The authors developed a wearable wireless sensor based on the simulation results and compared the SpO_2_ measurement result with the measurement from the commercial fingertip-type pulse oximeter that showed a high degree of correlation (~98.26%) between the measurements. 

Some researchers also worked on designing on-chip oximeters for SpO_2_ measurement. Tavakoli et al. [107] developed a low-power analog single-chip pulse oximeter fabricated in a 1.5 µm AMI BiCMOS n-well process. They used a conventional fingertip probe to measure the SpO_2_ level in blood of 11 subjects and compared the results with that of a commercial pulse oximeter. It has been observed that the result closely follows the measurements obtained from the commercial pulse oximeter having a standard deviation of ~1.5%. The chip has a dimension of 2.2 × 2.2 mm^2^ and only requires 4.8 mW of power, which according to the authors is more than an order-of-magnitude less than the power consumption of the best pulse oximeters available in the market. 

A multi-sensor chip was designed by Dunn et al. [97] that can be integrated into an electronic patch for measuring temperature, pulse and SpO_2_. The authors proposed a ring-shaped photodiode (PD) for reflectance oximetry that maximizes the gathering of backscattered light from the tissue, thus enabling much lower driving current for LEDs. The PD also contains an Au thermistor which facilitates measurement of skin temperature. With a current requirement of less than 10 mA, this multi-sensor oximeter chip is suitable for long-term monitoring purposes.

Other SpO_2_ monitoring systems such as forehead mounted sensor [108], electronic patch sensor [109] and phone camera based measurement systems [110] can be found in the literature. Table 7 presents the comparison of SpO_2_ monitoring systems discussed above.

### 2.6. Multi-Sensor Monitoring System

As discussed above, most of the systems were developed aiming to measure or monitor only a specific bio-signal or parameter, for example, only ECG and HR were monitored in [10,28,30,31,32,33]. However, it is necessary to monitor a set of physiological signs such as HR or pulse, BP, respiratory rate, and body temperature; often referred together as vital signs as well as oxygen saturation level in blood and GSR level in order to perform a better assessment of an individual’s health condition. Using parameter specific monitoring systems for each parameter is neither practical nor ergonomically sound for continuous and ambulatory monitoring. A network of multiple on-body sensors embedded in a wearable platform along with an on-body data acquisition and transceiver module can be a viable solution for multi-parameter monitoring.

**Figure 9:**
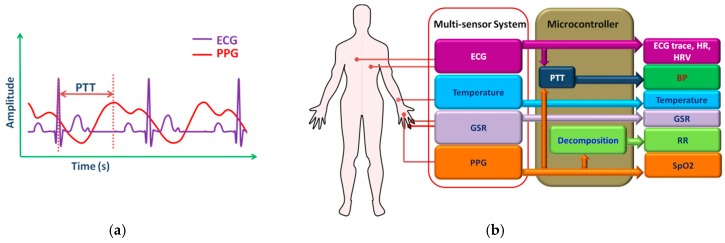
(**a**) Pulse transit time (PTT); (**b**) Four sensor health monitoring system.

From the discussion presented above, it is noted that a set of important physiological parameters can be measured and monitored by using four sensors: ECG, PPG, GSR, and temperature sensor. An ECG sensor measures ECG signal, HR, and HRV while GSR and temperature sensor measures skin conductivity and body temperature, respectively. PPG signal is generally used to determine the arterial oxygen saturation (SpO_2_) level. Many researchers exploited Moens–Korteweg or Bramwell–Hill relationship in order to estimate the BP from the pulse transit time (PTT) (Figure 9a), the time interval between the ECG and PPG signal peaks [111,112,113,114,115]. Furthermore, ECG and PPG signal can also be used to determine respiration rate (RR) [116,117,118,119] by employing signal decomposition techniques such as empirical mode decomposition (EMD) [117,118], principal component analysis (PCA) [118,119] or wavelet transform [120,121]. Figure 9b presents the concept of a four sensor monitoring system. 

## 3. Textile-Based Wearable Sensors

**Figure 10:**
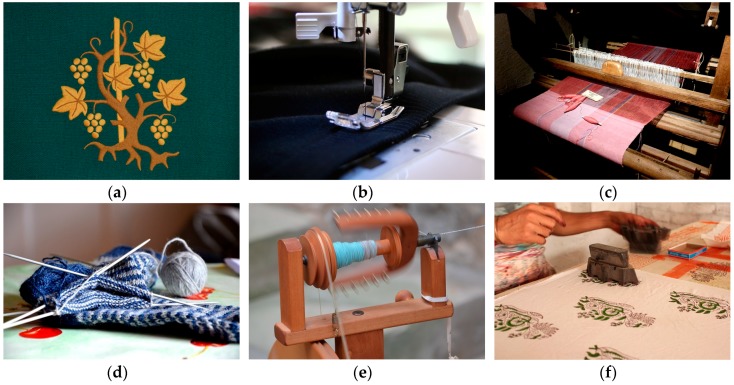
Different textile/fabric manufacturing technologies. (**a**) Embroidery; (**b**) Stitching; (**c**) Weaving; (**d**) Knitting; (**e**) Spinning; (**f**) Printing. Image source: https://pixabay.com under Creative Commons CC0.

Smart textiles associated with healthcare include sensors, actuators, communication, computing, and electronic systems that are made of textile or are suitable for embedding into textiles thus enabling unobtrusive and comfortable means of monitoring physiological signals of the individuals [122,123]. It makes use of conventional fabric manufacturing techniques such as weaving, knitting, embroidery, and stitching to realize or integrate sensing materials in clothes. Advanced fabrication methods, for example, inkjet-printing, coating, lithography, chemical vapor deposition (CVD) are also used in order to achieve high performance in terms of noise and sensitivity. Figure 10 shows different conventional textile manufacturing technologies. The active or sensing material is usually built on a substrate and can either be in direct contact with the body surface or remain encapsulated in a fabric-based layer [124,125,126].

### 3.1. Textile Electrodes

Smart textiles can be used to develop wearable on-body electrodes in order to measure electro-physiological signals such as ECG, electroencephalography (EEG), GSR, and electromyography (EMG). Textile based electrodes were reported to be as reliable as the traditional wet gel Ag-AgCl electrodes [127,128]. Textile electrodes can be classified into two basic categories: active and passive. Passive textile electrodes sense electrical properties from the skin surface. It can be used to monitor cardiac or muscle activities by sensing potential fluctuations caused by the heart or muscle. They also have applications in GSR measurement where the change in the skin conductivity due to sweating is detected by attaching electrodes on the body surface. Traditional electrodes use adhesive and conductive gel to affix them to the skin. It requires skin preparation such as shaving and cleaning the attachment site. In addition, the conductive gel may cause irritation, allergic reactions, or inflammation [10,129,130]. Furthermore, the gel dries out with time causing degraded signal quality. Although wet electrodes provide superior signal quality, they are not suitable for wearable and long-term monitoring system [10]. 

On the other hand, dry electrodes do not use adhesive or conductive gel and are usually biocompatible. Owing to their “dry” nature, they are more suitable for long-term monitoring and are being used extensively in textile-based health monitoring systems [129,130,131,132,133,134,135]. However, dry electrodes suffer from very high electrode-skin impedance and thus are more vulnerable to noise and motion artifacts compared to the wet electrodes. Active electrodes often incorporate a high input impedance preamplifier that reduces the impact of noise and motion artifacts by reducing the electrode-skin impedance [131,132,136]. This also helps to reduce the impedance mismatch between the electrodes resulting in lower differential common mode voltage, which may otherwise cause signal saturation. Some active textile electrodes can stimulate muscle or nerve cells by applying an electrical current to the underlying tissues. This technique of muscle and nerve activation, commonly referred to transcutaneous electrical nerve stimulation (TENS) is widely applied in rehabilitation and therapeutic applications [133,134,135] such as chronic and postoperative pain management. 

Textile electrodes can be realized by integrating prefabricated electrodes into finished garments by simply stitching them at suitable locations on clothes. They can also be developed by directly depositing conductive layers on the fabric. The conductive layers can be formed on the surface of the fabric by depositing nano-fibers [137,138,139] using electrodeposition method or by applying a conductive layer with the help of screen printing [140], sputtering, carbonizing and evaporation [141].

Although conductive coating on the surface of the fabric results in superior conductivity, the performance may deteriorate with time, especially after a number of wash cycles. Another attractive technique of textile electrode fabrication is weaving or knitting [136,140,141] garment fabrics using conductive yarn. The conductive yarn can be made of metal filaments [141,142,143], conductive nano-filaments [144] or produced by applying a metal coating on fibers such as cotton [136,137,140], nylon [141], Kevlar or polyester [136,142,143,144,145]. Nano-fibers can be grown by the electrospinning method [138] whereas metal coating on the fiber is formed by employing chemical deposition process such as polymerization [146], electroless plating [141,145], electroplating [147] and sputtering [141,144]. Table 8 presents the summary of several textile electrodes reported in the literature. 

### 3.2. Textile Based Temperature Sensors

Rectal thermometer is usually the most accurate method of measuring body temperature. However, this method is invasive, time-consuming and require private arrangements, thus making it unsuited for continuous monitoring purposes. A tympanic thermometer measures temperature from the tympanic membrane of the ear by placing a probe in the auditory canal which is not convenient for long-term use. Besides this, the ear canal needs to be properly cleaned as earwax can decrease the measurement accuracy [148]. Oral thermometers are also known to provide reliable readings when measurement is taken from the sublingual pocket. Axillary (armpit) temperature measurement is more convenient compared to the above-mentioned methods. This method is sometimes considered unreliable for core body temperature estimation owing to the absence of any primary blood vessel at this site [149]. The measurement can also be affected by the environmental temperature and perspiration. However, both oral and axillary measurement techniques restrict movement of certain body parts and thus, are not suitable for continuous monitoring. 

The normal core body temp of a person remains within a narrow range of 36 °C–37.5 °C, although it can vary by few degrees due to environmental influence, diseases or physical activities. The skin temperature differs from the core body temperature by as much as 2.5 °C [150]. Therefore, the temperature sensors used for body temperature measurement need to cover a temperature range from 35 °C–40 °C. It is also important to ensure measurement accuracy that, otherwise can impact the diagnosis and treatment. Typically, an accuracy of 0.1 °C is desired [151]. Textile based flexible temperature sensors can be useful for measuring human body temperature in a wearable platform. They can be fabricated from fiber or single yarn using conventional textile manufacturing technologies such as weaving, knitting, embroidery, and printing. Temperature sensors fabricated on flexible substrates can also be incorporated into textiles. Whatever be the case, higher accuracy, linearity, sensitivity, and fast response time within the range of 35 °C–40 °C are critical for the temperature sensors. Apart from that, selection of proper encapsulation material is also important in order to protect it from external mechanical and environmental impacts.

Based on their operation principles, textile-based temperature sensors can be categorized as thermocouples and resistance temperature detectors (RTD). Thermocouples exploit the Seebeck effect that develops a corresponding potential difference across the junctions of two dissimilar metals due to the temperature difference between the junctions. The sensitivity of thermocouples usually ranges from ~10 µV/°C to 70 µV/°C [152,153]. Various textile thermocouple structures using different conductive and metal coated non-conductive threads were reported in [154]. Among different pairs of materials, thermocouple developed with steel knitted fabric-constantan wire pair exhibited the highest sensitivity (41.4 µV/°C). However, thermocouples exhibit non-linear relationship between potential variation and temperature as well as very low sensitivity that is not suitable for human body temperature measurements [155,156]. 

The RTDs exploit the materials’ temperature dependence of electrical resistivity to determine the temperature. RTDs offer higher accuracy, sensitivity, and shorter response time as well as linearity with temperature. Textile based RTDs can be realized by simply embedding metal or conductive threads with a strong TCR (temperature coefficient of resistance) into fabric using conventional textile manufacturing technology such as knitting [157], weaving [158], and embroidery [159]. Many researchers are also working to develop flexible temperature sensors for textile applications. Such sensors were fabricated by depositing sensing materials on flexible polymer substrates such as Kapton [158,160,161,162], polydimethylsiloxane (PDMS) [163] or paper substrates [164] using screen printing [160,162,165], inject printing [161,164], CVD [163] techniques. Metals such as silver, platinum, nickel or metal alloys were used widely as the sensing material, whereas some researchers exploited nano-materials such as carbon nanotube [162], graphene nano-wall [163] as the temperature sensing element. Hongqiang et al. [166] presented an optical sensing approach for body temperature measurement. They used a distributed Bragg reflector (FBG) that reflects light of specific wavelengths and transmits at other wavelengths. The FBG was encapsulated with a polymer substance which was later weaved into the textile. The authors mathematically analyzed the transmission of heat from skin to the environment via FBG sensors and employed a weighted coefficients model to estimate the body temperature. The authors reported achieving high measurement accuracy (~±0.18 °C) within the range of 33 °C–42 °C. Table 9 presents the summary of textile based temperature sensors discussed above.

### 3.3.Textile Sensors for Activity Measurement

Most researchers have used MEMS based inertial sensors such as accelerometers, gyroscopes or magnetic field sensors or their combinations in order to measure the signal corresponding to human locomotion. They are mounted on small PCB boards, which are usually embedded in belts, elastic bands, and Velcro straps. MEMS-based motion sensors are cheap and small in size. Having good sensitivity, accuracy, and low power features, they are suitable for long-term and real-time activity monitoring systems. However, the rigid PCB boards may feel uncomfortable to some users. Rajdi et al. [167] fabricated a MEMS accelerometer on cotton cloth for measuring pelvic tilt angle. The accelerometer exploited the piezoresistive effect of the conductive Ag nanoparticles that was patterned on the textile by stamping and ironing. When the tilt angle varies, the cantilever beam structure of the accelerometer experiences mechanical stress that eventually changes the resistance of the conductive material in a linear fashion. The authors reported a strong positive correlation between relative resistance change and the strain applied on the textile-based accelerometer.

Flexible and stretchable strain sensors were also used by many researchers in textile-based activity monitoring systems. Strain sensors measure the physical deformation by changing its electrical characteristics such as resistance and capacitance in response to mechanical stress. The strain sensors for textile applications need to be highly flexible, stretchable, and durable. In addition, high sensitivity, and fast response/recovery time are critical for real-time activity detection [152,153].

Amjadi et al. [168] presented highly flexible strain sensors based on the nanocomposite of Ag nanowire mesh, which was embedded between PDMS layers. The piezoresistive strain sensors exhibited high sensitivity with a tunable gauge factors (GF) in the ranges of 2 to 14, stretchability up to 70%, and fast response. The authors demonstrated the usability of their designed strain sensors by detecting the motion of the fingers in real-time using a glove that had five sensors embedded in its five fingers. Similarly, Lee et al. [169], developed a highly sensitive, stretchable, and durable strain sensor using a thin film of Ag nano-particles. The film was printed on a PDMS membrane by the direct transfer process. When a mechanical stress is applied, it creates micro-cracks in the film, causing a change in the resistance of the sensor. The sensor showed fast response (~1 s) and recovery (~0.5 s) times. The devices were embedded in a glove to detect the activity (bending/relaxation) of the finger joints. Shyr et al. [170] developed a textile strain sensor that was implemented in a gesture sensing device to measure the flexion angle of elbow and knee movements. The strain sensor was fabricated from elastic and conductive webbing. The conductive yarns were made from carbon particles coated polyamide fiber twisted with polyester yarn whereas the elastic yarns were made from Lycra fiber wrapped with two polyester yarns. The elastic conductive webbing exhibited good linearity in resistance with flexion angles.

Zhang et al. [171] developed a textile-structured flexible strain sensor by using conductive fiber. The authors exploited the variation in fiber-fiber, yarn-yarn loop, and fabric-fabric contact resistances with strain in a textile type structure. They used metal and carbon fibers as the sensing material due to their good physical and electrical properties. The sensors exhibited good linearity within a large strain range and also achieved high gauge factor and sensitivity. However, the sensitivity of the sensor can be affected by the shape, width, and density of the fibers. The strain range can be improved by including elastic fibers in the structure. Another interlock based textile structure was presented in [172] where the authors knitted a strain sensing fabric from elastomeric yarns. A series of loops were embedded in this fabric by knitting a silver coated polymeric yarn in it. The interlock structure has the advantage of having higher dimensional stability, and enhanced sensor repeatability. The loop structure of the sensing conductive yarn also helps to minimize structural deformation over long term use. The authors used three different sensors fabric yarns with different density, tightness factor, and input tension and observed different linear range and gauge factor for different sensors. However, knitting with metallic fibers or yarns may cause structural damages to the textile due to excessive friction [173]. The knitted fabric also can be uncomfortable due to added stiffness of the metallic fibers or yarns. An electrically conductive and flexible all-polymeric fiber was presented in [174]. Polymer fibers offer high flexibility and less friction; and thus, they increase the lifetime of the structure. The fiber was developed from PU/PEDOT:PSS by using the wet-spinning method. Up to four such fibers were knitted with commercial Spandex yarn. It was observed that the sensitivity of the strain sensor increased with the number of PU/PEDOT:PSS fibers. The sensor exhibited stable sensing performance upto 160% of applied strain.

A few researchers investigated textile-based optical sensors for activity monitoring. An optical Fiber Bragg Grating (FBGs) based knee monitoring system was proposed in [175]. A single optical Fiber Bragg Grating (FBGs) sensor encapsulated with a polymer foil was integrated into an elastic knee band. The flexion-extension movement of the knee causes strain variations resulting deflection in the resonance wavelength of the FBG. They also integrated the FBG sensors in gloves and Velcro straps in order to demonstrate the usability of FBG sensors in detecting finger movements and heart, respiration rate. Instead of optical power, the FBG sensor performed measurement based on the wavelength thus was less sensitive to external noise and fluctuations in the optical source. The system exhibited high sensitivity, stability, and measurement accuracy. Krehel et al. [176] designed an optical fiber based flexible force sensor that could be potentially be integrated into textiles. The optical fiber was fabricated from a flexible copolymer containing silicon and polyurethane. In the presence of an external force, the optical fiber experiences an elliptical deformation along the plane of its cross section. This deformation causes increased deflection of light within the fiber resulting reduced intensity of light at the output. The force, therefore, can be estimated from the output light intensity. The sensor is flexible and can be integrated into textile for detecting moderate to strong forces corresponding to, for example, limb motion, and respiratory rate. The sensor is sensitive to strain, bends, and temperature which cause inaccuracies in the measured data. Table 10 presents the summary of textile-based strain sensors.

## 4. Communication Technologies for Wearable Systems

The physiological signals measured by the on-body sensors need a two-stage communication to transmit the data to the remote healthcare server. In the first stage, a short-range communication protocol is employed to transmit the measured data to a nearest gateway node such as PDA, smartphone, computer, custom-designed FPGA, or a microcontroller-based processing board. The gateway is responsible for advanced data processing, display, and the next long range communication stage, where the processed signal is transmitted to a distant server placed in a healthcare facility.

The data can be transmitted over the internet or cellular communication network. Currently, most cellular networks offer seamless access to the internet through General Packet Radio Service (GPRS), Enhanced Data GSM Environment (EDGE), 3G, High Speed Packet Access (HSPA), Long-Term Evolution (LTE) services [177,178,179]. However, it is essential to implement strong encryption and authentication technology in order to ensure a secure transmission channel over the long range communication medium for safeguarding of personal medical information [180,181,182].

In the case of short-range communication, the sensors can communicate to the gateway directly over a wireless medium. Alternatively, the sensors can form a body sensor network (BSN), a star network topology and send data to the central BSN node. The BSN node can send data to the gateway after performing some processing. The on-body sensors and the BSN node could communicate by using wired or wireless medium. However, wired connections can hinder the users’ mobility and may cause frequent failed connections. Thus, they are not suitable for wearable and long-term monitoring systems. A good option is to use conductive fabric yarns as the alternative conductive medium. These fabrics can be easily integrated into clothing to communicate with textile embedded sensors [183,184,185]. As discussed earlier, the conductive fabric can be fabricated using conventional textile technologies such as weaving, stitching, embroidery, and printing. But conductive fibers do have a problem due to their low durability and washability that may lead to poor or failed connectivity after a long period of use. Therefore, wireless technology can be adopted as the most viable and reliable alternative for short-range communication. In this section, we will present a brief overview on short range wireless communication protocols that have been used in recent researches.

Bluetooth is a popular low power RF communication technology that has been widely used in devices such as laptops, smartphones and fitness trackers for short range data communication [186,187]. It uses the 2.4 GHz frequency band in the industrial, scientific and medical (ISM) radio spectrum and transmits signal over 79 designated channels using the Frequency Hopping Spread Spectrum (FHSS) method. The FHSS method is less susceptible to noise and interference and also offers highly secured data transmission. One master device can communicate with seven slave devices thus, forming a star type network structure based on Bluetooth connectivity (Piconet). The master defines the clock and hopping sequence for the whole Piconet. The Bluetooth technology can support a data rate of ~3 Mbps depending on the modulation schemes, although the maximum throughput may only reach ~2.1 Mbps. For general applications, the transmission distance typically ranges from 1 m to 10 m. An ultralow-power version of Bluetooth technology, named as Bluetooth low energy (BLE) or Bluetooth V4 was later introduced for portable and wearable devices with limited battery capacity [15,187]. BLE uses the same frequency band as classical Bluetooth technology but hops over 40 channels with each channel having a bandwidth of 2 MHz. BLE, as the name indicates, offers low power (~10 mW) wireless connectivity and, thereby is a strong candidate for short range communication in long-term monitoring systems. 

Another popular and open wireless standard for low power and low-cost communication within short range is ZigBee [15,17]. It operates in the unlicensed 2.4 GHz (worldwide), 915 MHz (Americas and Australia) and 868 MHz (Europe) frequency bands of the ISM spectrum and transmits data over sixteen, ten and one channels, respectively. The 868 and 915 MHz bands use the binary phase-shift keying (BPSK) modulation whereas offset quadrature phase-shift keying (OQPSK) is used at the 2.4 GHz band. Unlike Bluetooth and BLE that only supports peer-to-peer (P2P) and star topologies, ZigBee devices can be connected using P2P, star, tree and mesh network topologies. Prior to transmitting a packet, the ZigBee protocol first assesses the communication link by using CSMA/CA (carrier sense multiple access with collision avoidance) protocol or by sending beacons to other nodes in the network. The transmission range of ZigBee standard is limited to within 10–20 m for indoor applications mostly because of its low output power and also the presence of high dielectric materials. However, the range can increase up to 1500 m with no obstacles in the line of sight. The data rate is much lower compared to the Bluetooth technology and can reach maximum 250 kbps for the 2.4 GHz band. However, the low power requirement of ZigBee standard leads to extended battery life that is advantageous for long-term health monitoring applications, although the lower data rate may impose limitations on the number of sensors, number of simultaneous measurements, and data buffering in a multi-sensor network. Faster RAM along with efficient first-in-first-out (FIFO) and data compression algorithm need to be implemented in the central BSN processing hardware. 

ANT is a proprietary protocol stack designed for ultra-low-power, short-range wireless communications in sensor networks, especially for health and fitness monitoring systems [19,188]. It ensures low power consumption by using low data rate, shorter delay cycles, and deep-sleep mode and can operate for longer periods of time, for example, it can run a year on a 250 mAh coin cell battery. Similar to other wireless protocols presented above, it also operates in the 2.4 GHz ISM band. It uses TDMA (time division multiple access) to communicate with multiple nodes over a single 1 MHz channel. It can switch channels if any interference occurs. ANT can be distinguished from other wireless protocols by its unique feature in which it acts as a master for one channel while simultaneously serving as a slave for another channel. Like ZigBee, ANT supports multiple network topologies and also ensures coexistence with neighboring ANT nodes using adaptive isochronous network technology. The maximum data rate achieved by ANT systems ranges from 20–60 kbps and there is a trade-off between data rate and low power consumption. A recent advancement in ANT protocol, ANT+, uses application specific 'device profiles’ to communicate between two devices. For example, the applications can be one of vital signs monitoring, walking speed and distance monitoring, or fitness monitoring and "device profiles" are the set of network rules, parameters, data format specific to a particular application. Furthermore, the ANT+ protocol has the advantage from interoperability with other ANT+ devices having the same device profile.

The medical implant communication service or MICS, is a short-range, ultra-low power wireless technology that was developed to communicate with implanted medical devices such as cardiac pacemakers, defibrillator, and neuro-stimulators [189,190]. It operates within the frequency band of 402–405 MHz with 300 kHz channels. This frequency band offers good signal propagation characteristics in the human body that makes it suitable for implantable devices. MICS uses the listen-before-talk (LBT) protocol to assess the link before starting transmission. In the case of any interference, MICS switches to a different radio channel and listens again. The MICS system has a typical transmission range of ~2 m and consumes as little as 25 µW of power. However, due to limited availability of commercial MICS devices along with some networking constraints [191], this technology has not been used much in wearable systems. 

There are a few other wireless technologies for short-range communication such as Infrared Data Association (IrDA), Ultra-Wide Band (UWB), Radio Frequency Identification (RFID), Near Field Communication (NFC) and WiFi are also available. IrDA was one of the most popular wireless technologies for very short-range communication (<10 cm) because of their high data rate. However, the communicating IrDA devices need to maintain line-of-sight for transmission that makes it infeasible for wearable monitoring systems. The UWB operates in the wide frequency spectrum of 3.1–10.6 GHz and uses short Gaussian impulses or multi-band orthogonal frequency-division multiplex (OFDM) signal for communication. It offers very high data rate at a very low power spectral density, which protects it from possible interference with other radio waves. Although UWB technology has promising applications in medical monitoring and imaging [192,193], the complexity and limited availability of commercial UWB systems makes it impractical for wearable systems. RFID is another popular wireless technology that is widely used primarily for tracking and identification purposes. RFID technology uses different frequency bands including the ISM band. A reader or interrogator sends a signal to a tag or label that is attached to an object to be identified [194,195]. On the other side, NFC is a low cost, low power wireless technology with a communication range of ~20 cm. It operates in the frequency band of 13.56 MHz and is compatible with passive RFID technology that comes at the price of increased power consumption. NFC only supports P2P communication between two devices, so it is not appropriate for wearable BSN systems [196,197].

Finally, WiFi, due to its extremely high power consumption and complex configurations, is inefficient for long-term monitoring systems where longer battery life is indispensable. Table 11 presents the key features of currently available wireless technologies and their usage in current wearable health monitoring systems.

## 5. Conclusions and Research Challenges 

In this paper, we have presented a state-of-the-art survey on physiological parameters and activity monitoring systems developed in a wearable platform. The primary purpose of a wearable health monitoring system is to allow people to lead independent and active lives in their familiar home environment while ensuring continuous, non-invasive, non-intrusive, and seamless surveillance of their health and physical well-being. The enormous development of technology in the past few decades leads to manufacturing and use of miniature, low-power, low-cost sensors, actuators, electronic components, and powerful computers that paves the way to non-invasive, non-intrusive, and continuous monitoring of an individual’s health condition at a very low-cost. 

Continuous monitoring of health status can provide comprehensive information about individuals’ health status over a period of time. The wearable sensors and actuators, coupled with the advanced information and communications technologies have opened the window to a new era of cost-effective remote healthcare services. The systems can include monitoring and data analysis as well as predictive algorithms, which can potentially make the prognosis of certain diseases with a higher degree of confidence, thereby leading to early diagnosis and treatment. In the case of any potential health issue is detected, the system can raise an alarm and notify the persons concerned or the healthcare services via secure wireless media such as the internet and the cellular network so that immediate medical intervention can be initiated. Incorporation of smart textiles technologies such as textile-based interconnections for sensors in wearable healthcare systems could lead to more comfortable, non-intrusive platforms for health monitoring. 

In addition to the significant advances in the past decade, there are challenges that require further research and development in order to improve the performance of the heart monitoring system. 

First, the bio-potential (ECG, EDA) measurement systems very often suffer from low signal-to-noise ratio (SNR) that primarily evolves from the noise induced by the movement of the user. Motion artifact (MA) can be minimized by employing flexible electrodes or high input impedance front end amplifiers. It is also possible to improve the SNR by exploiting signal processing techniques such as adaptive filtering, empirical mode decomposition (EMD), independent component analysis (ICA) or time-frequency analysis. 

Second, the hardware and computation resource for the on-body central node of a multi-sensor BSN system can be a limiting factor for seamless connectivity and data handling. The central processing node of the BSN network exchanges data with the on-body sensors as well as the home gateway, and sometimes performs limited processing. Therefore, a robust and efficient algorithm is required for the central BSN node to optimize its performance. In addition to that, an efficient data compression algorithm needs to be implemented in the central node in order to deal with a large volume of data and transmit them to the nearest gateway. 

Third, a key concern for the wearable healthcare system is associated with the privacy and security of the sensitive medical information of the user. More efforts are needed in order to develop algorithms to ensure highly secured communication channels in existing low power, short range wireless platforms.

Fourth, low power consumption and high energy efficiency are critical for long-term monitoring systems. Power requirement of the system can be satisfied by using low power components, more efficient batteries or by employing energy harvesting techniques. Battery lifetime can also be improved by ensuring ‘sleep and wake up’ of the sensors in a timely fashion without disrupting the desired measurement frequency. 

Fifth, in order to achieve widespread acceptance among the people, the systems need to be affordable, easy-to-use, un-obtrusive, and inter-operable among various computing platforms. A minimum numbers of electrodes, sensors need to be used yet not to lose the most important clinical information. Therefore, more research and development efforts are needed to enhance the systems’ ease-of-use. 

Sixth, we have also presented a brief review on textile-based sensors highlighting their applications in sensing physiological signs. They can be fabricated by using conventional textile technologies such as knitting, weaving, embroidery or printing. Textile based sensors, that is smart textiles, have great potential in wearable monitoring systems. For example, textile-based electrodes and temperature sensors can be used for physiological measurements, whereas textile-based strain sensors can be exploited for monitoring HR, respiration rate, pulse as well as human activities. However, ensuring high signal accuracy, sensitivity, SNR and stability in a textile-based platform are the key design challenges. Further, more work is needed for the proper selection of sensing materials and embedding technique as well as stable sensor-skin interface to ensure superior sensor performance. In addition, durability and signal integrity of the sensors with time and washing cycles should also be improved while fabricating smart textiles for long-term health monitoring. 

We have discussed and compared various wireless technologies and assessed their feasibility in wearable health monitoring systems. Generally, the systems measure several physiological parameters from the human body and transmit them to a central node or main gateway. The gateway node processes and transmits the data to a healthcare personnel in a remote facility. However, more research and technology development is needed to ensure information privacy and data security, robust data compression algorithms, reliable communication link, and energy efficiency. 

## Figures and Tables

**Table 1:** Listing of some commercial products for monitoring physiological signs and activities.

Product Name	Monitored Parameters	Wireless Platform	Battery	
			Type	Life
Hexoskin^®^ Biometric^®^ Shirt	Heart rate (HR), HR variability, respiratory rate, number of steps, distance traveled, pace, maximal oxygen consumption, and calories burned.	Bluetooth	6–7 days (standalone) 14+ h (multi-training)	
Jawbone UP3™ Fitness Tracker	Sleep stages (REM, light and deep), HR, food and liquid intake, number of steps, distance traveled, running.	Bluetooth LE	Li-ion poly	7 days
Striiv^®^ Fusion Bio Fitness Tracker	HR, number of steps, distance traveled, calories burned, and sleep quality.	Bluetooth LE	Li-ion	5 days
Microsoft^®^ Band 2	HR, calories burned, sleep quality, food, and liquid intake, number of steps, elevation, climbing, running, biking.	Bluetooth	Li-poly	2 days
Fitbit Charge HR™ Fitness Tracker	HR, calories burned, sleep quality, food, and liquid intake, number of steps, elevation, climbing, running.	Bluetooth LE	Li-poly	5–7 days
Garmin vivosmart^®^ HR Fitness Tracker	HR, calories burned, sleep quality, number of steps, climbing, running, swimming.	Bluetooth LE, ANT+	Li-ion	5 days

**Table 2:** Comparison among cardiovascular monitoring systems.

Ref.	Proposition	Moni-Tored Signs	Electrode Type	Active Material	Electrode Size	Attachment Method	Wireless Connectivity	Accuracy	Signal Acquisistion Module			
									Size	Freq.	A/D	Bat. Life, Power
[28]	Sensorized T-shirt and textile belt	ECG, HR	Dry textile electrodes	Silver based conductive yarns		Snap buttons	Bluetooth LE		-	512 Hz	24 bit	-
[29]	Wearable mobile electro-cardiogram monitoring system	ECG, HR, location	Dry foam electrode	Ni/Cu coated compressed urethane polymer foam	14 mm × 8 mm × 8 mm		Bluetooth v2.0, and GSM	99.51% correlation with prerecorded ECG data, QRS detection accuracy ~98.14%	4 cm × 2.5 cm × 0.6 cm	512 Hz	12 bit	33 h, 1100 mAh Li-ion battery
[10]	Wireless, portable capacitive ECG sensor	ECG, HR	Capacitive electrode with cotton insulator		33 mm × 33 mm × 2 mm	Woven under a stretchable belt	ANT		45 mm × 60 mm × 9 mm	500 Hz	10 bit	15 h with 256 mAh 3 V Li battery
[30]	Use of flexible capacitive electrodes for reducing MA	ECG, HR	Flexible capacitive electrodes	Ni/Cu coated foam (polyolefincovered by polyurethane)	300 mm × 20 mm × (1.1 ± 0.2 mm)	Integrated into a chest belt	Bluetooth	Upto 91.32% QRS detection at 7 km/h walking speed	-	256 Hz	-	-
[31]	Common Electrode-FreeECG monitoring System	ECG, HR	Active capacitive electrodes	Copper layer	5 cm × 3 cm	Adhesive tape	-		-	2 kHz	24 bit	-
[32]	HR monitoring from pressure variance in ear canal	HR	Piezo-electric film sensor		3.5 mm × 3.5 mm	Earpiece like device	2.4 GHz RF	Sensitivity 97.25%, PPV 97.18%.	15 mm × 17 mm	100 Hz	12 bit	Coin-cell battery
[33]	Heart Rate Monitoring with pressure sensor	HR	Piezo-resistive pressure sensing	C black/silicone rubber nanocomposite encapsulated in conductive FCCL films	15 mm × 30 mm	Embedded in elastic belt	-	Accuracy > 97%	-	-	-	-

**Table 3:** Features extracted from motion signal.

Spatial Domain	Temporal Domain	Frequency Domain	Statistical Domain
Step length	Double support time	Spectral power	Correlation
Stride length	Stance time	Peak Frequency	Mean
Step width	Swing time	Maximum spectral amplitude	Standard deviation
RMS acceleration	Step time		Covariance
Walking speed	Stride time		Skewness
	Cadence (steps/min)		Kurtosis
			Energy

**Table 4:** Comparison among activity monitoring systems.

Ref.	Proposition	Feature Extraction	Classification Method	Sensors	Sensor Placement	Com. Tech.	Detection	Accuracy	Power Req.
[44]	Activity and gait recognition system on a smartphone	Fixed set of features	Support Vector Machine (SVM), Bayes network, and Random Tree	Accelerometer is embedded in smartphone			Different walking speed	>99%.	
[45]	In-home, fine-grained activity recognition multimodal wearable sensors	Fixed feature set	Conditional random field (CRF)	Smartphones’ (Samsung Galaxy S4) onboard sensors (accelerometer, gyroscope, barometer, temperature and, humidity sensor), along with Gimbal Bluetooth beacons	Waist, lower back, thigh, and wrist	USB	Walk and run indoors, use refrigerator, clean utensil, cook, sit and eat, use bathroom sink, move from indoor to outdoor, move from outdoor to indoor, walk upstairs, and walk downstairs, stand, lie on the bed, sit on the bed, lie on the floor, sit on the floor, lie on the sofa, sit on the sofa, and sit on the toilet	19 in-home activities with >80% accuracy	
[46]	Wearable device based on a 9-DOF IMU	Fixed set of features		Accelerometer, gyroscope, and magnetometer	Limb or trunk	Bluetooth	Balance hazards, balance monitoring for fall prediction	High correlation	Streaming ~6 h Logging > 16 h
[47]	Algorithm development	Time-Frequncy domain analysis	Hidden Markov Model	3-axis accelerometer, 3-axis gyroscope	Chest	USB	Walking, running, ascending upstairs, descending downstairs and standing	~95%	
[48]	A real-time, adaptive algorithm for gait-event detection			Two inertial and magnetic sensors ( 1 IMU = 1 accelerometer, 1 gyroscope)	External part of both shanks		Gait events: Initial Contact (IC), End Contact (EC) and Mid-Swing for both right and left leg while walking at three different speed	F1-scores 1(IC, EC), 0.998 (IC) and 0.944 (EC) for stroke subjects	
[49]	Recognition method for similar gait action	Inter-class relation Ship	Support vector machine, K-nearest neighbor	3 IMUs (each IMU: 1 tri-axis accelerometer,1 tri-axis gyro)	Fixed at the back, left, and right waist		Walking on flat ground, up/down stairs, and up/down slope	~93% average	
[50]	Stochastic approximation framework	Fixed set of features	K–means and Gaussian Mixture Models	Accelerometer	Belt-like strap around the waist		3 intensity level of walking: 93.8%; 3 intensity level of running 95.6%		
[51]	Power-aware feature selection for minimum processing energy	Minimum cost feature selection by using a redundancy graph	K-nearest neighbor	6 IMUs (each IMU has one three-axis accelerometer and a two-axis gyroscope)	Waist, right wrist, left wrist, right arm, left thigh, right ankle	BSN	Switching between stand and sit, sit and lie, bend to grasp, rising from bending, kneeling right, rising from kneeling, look back and return, turn clockwise, step forward and backward, jumping	30% energy savings with 96.7% accuracy	
[52]	Parameter optimization strategy for phase-dependent locomotion mode recognition	Fixed set of features		2 IMUs, 2 pressure insoles (each having 4 pressure sensors)	IMUs on the shank and the shoe, pressure sensors insole		Walking, up/down stairs, and up/down slope, passive mode	88%–98%	
[53]	Electronic insole for wireless monitoring of motor activities and shoe comfort	Fixed set of features		Humidity and temperature sensors, accelerometer and 4 pressure sensors	Insole	ZigBee	Foot accelerations, orientation in space, temperature and moisture data		10 h of data logging
[54]	Shoe-based activity monitoringsystem (smartshoe)	Fixed set of features	Support vector machine, multilayer perception (MLP)	Five pressure sensors (PS) and one 3-D accelerometer	PS on insole and accelerometer on heel of shoe		Sit, stand, walk, ascend stairs, descend stairs and cycling	99.8% ± 0.1% with MLP	
[55]	A wearable device for monitoring daily use of the wrist and fingers	Fixed set of features	K-means	2 tri-axial magnetometers	Watch-like enclosure worn on the wrist and a small neodymium ring worn on the index finger		Finger and wrist movement	92%–98% with a 19%–28% STD	20.5 mA at 3.3 V
[56]	Combined kinematic models to estimate human joint angles	Unscented Kalman filter		3 IMUs	Upper arm, forearm, and wrist		Shoulder internal/external rotation; flexion/extension of shoulder, elbow, and wrist, supination/pronation of forearm, wrist twist	Average RMS angle error ~3°	
[58]	Wearable device with automatic gait and balance analyzing algorithms for Alzheimer patients (AP)	Fixed set of features		3 IMUs (each IMU has a 3-d accelerometer, a uni-axial gyroscope, and a biaxial gyroscope	On feet for gait analysis on waist for balance analysis		Gait parameters and balance		30 mA at 3.7 V
[59]	IMU based fall Detection system	Madgwick orientation filter		Accelerometer, gyroscope, and magnetometer	Waist	Bluetooth	Backward fall, forward fall, lateral left fall, lateral right fall, syncope	Accuracy: 90.37%–100% Sensitivity: 80.74%–100%	15 mA–34 mA using 3.7 V

**Table 5:** Body temperature monitoring systems.

Ref.	Proposed Device	Principle	Measured Parameters	Used Device for Measurement	Location	Wireless Connectivity	Performance Evaluation	Accuracy
[66]	Kalman filter based body temp. estimation model	Temperature variation with HR	HR, skin temperature	Ag/AgCl gel electrodes	Chest	-	Compared with data from ingestible temperature capsule	RMSE: 0.40 °C
[68]	Wireless, dual channel body temp measurement system	Mean of measurements from two ear canals	Core body temp	Digital temp sensor DS18B20	Ear canal	Bluetooth		±0.1 °C
[69]	Wearable wireless temperature monitoring	Two-point calibration	Circadian rhythms, Skin temp	MF51E NTC thermistor	Skin	RF (Tyndall node) over Body sensor network (BSN)	Compared with data from a thermometer	0.02 °C
[71]	Embedded NTC temperature sensor and conductive textile wires in a belt made with soft bamboo		ECG, skin temperate	NTC Mon-A-Therm 90045 and Shieldex^®^ Silver Plated Nylon yarn	Skin	-	Compared with data from the NICU sensor connected to the Solar^®^ 8000M patient monitor	±0.1 °C
[72]	Wireless body temperature monitoring		Skin temperature	LM35	Hand	ZigBbee and WLAN		±0.25 °C
[75]	RFID sensor chip in 0.35-μm CMOS standard process	Temperature dependence of the frequency of ring oscillator				Tag and reader communicate at 868 MHz	Measurement was performed in a climate chamber	~±0.1°C Resolution: 0.035°C
[76]	Epidermal-like RFID tag made on a Poli-caprolactone membrane	Re-tunable epidermal tag	Skin temperature	EM4325	Abdomen	Tag and reader communicate within a band of 780–950 MHz	Compared with data from PT104 thermocouple	±0.25 °C
[77]	Deep body temperature measurement system embedded in a neck pillow	Embedding 1 Dual-heat-flux, 2 double-sensor in neck pillow	Core body temperature		Around neck	-	Compared with data from infrared thermometer (thermoscan IRT 4520)	-
[79]	Heater-less deep body temperature probe	Dual-heat-flux method	Core body temperature		Forehead	-	Compared with data from zero-heat-flow thermometers	Correlation: 97%

**Table 6:** GSR monitoring systems.

Ref.	Proposition	Electrode Type/Device	Measurement Location	Wireless Connectivity	Size	Sampling Rate	A/D	Battery Life/Power Req.	Evaluation	Accuracy
[85]	A small wristband for unobtrusive and continuous EDA measurements during everyday activities	Ag/AgCl electrodes	Dorsal forearms	2.4 GHz transceiver module (nRF2401)	70 mm × 70 mm × 20 mm	32 Hz	12 bit	1199 mAh, 3.7 V LiPo	Measurement compared with commerecial system.	overall correlation: 93%–99%
[86]	An ambulatory device for measuring HR, GSR, and skin temperature	Arduino based e-textile lilypad platform (SHT15 for T measurement)		Not implemented				Supply voltage: 2 V to 5 V		
[87]	Highly wearable and reliable galvanic skin response (GSR) sensor	flexible dry polymer foam Ni/Cu	Back	Bluetooth	42.5 mm × 38.5 mm		10 bit		compared thesignal with a finger reference GSR	average Correlation: 76.8%
[88]	Wearable multi-sensor device for real-time biofeedback and data acquisition	Ag electrodes		Bluetooth LE	4 cm × 4 cm	4 Hz		38 h of operation	resolution 900 pS between 0.01 µS and 100 µS	
[89]	A pervasive and unobtrusive system for sensing human emotions	Commercial Shimmer GSR sensor	Finger	Bluetooth	65 mm × 32 mm × 12 mm	10 Hz		450 mAh Li-ion battery	Classification of 4 emotions with ~80% of accuracy (amusement, fear, sadness, and relaxation)	
[90]	Distinguishing stress from cognitive load in an office environment by EDA	Dry Ag/AgCl electrodes	left index and middle fingers	Bluetooth	41 mm × 67 mm	16 Hz		Power consumption: 182 mW	Investigated 6 classifiers to discriminate cognitive load from stress	Accuracy 82.8% (max), achieved by LDA
[91]	Use of wearable sensors and wireless technology to measure the autonomic function and stress level in the ambulatory setting	Ag/AgCl electrodes in Shimmer Platform	Palm of non-dominant hand	Bluetooth		30 Hz		GSR preconditioning circuit consumes 60 µA		
[93]	A wearable device for predicting blood pressure (BP) and cardiovascular dynamics	Ag/AgCl electrodes	Fingers or opposite sides of palm	Bluetooth		1280 Hz, averaged over 32 samples: results 40 Hz	10 bit	10 h with 9 V battery, 220 mA with Bluetooth	correlation with pulse pressure with GSR	R2 value for PP: 0.923, SBP: 0.801

**Table 7:** SpO_2_ monitoring systems.

Ref.	Proposition	Principle	Measured Parameters	Sampling Rate	Size	Power/Current Req.	Wireless Connectivity	Performance Evaluation	
[97]	Ring shaped backside silicon p-n photodiode	Transmittance oximetry	Temperature, Pulse, SpO_2_	8 kHz	Radius = 3.68 mm width = 0.78 mm	<10 mA		Quantum eff. = 62% Reverse current density = 55 nA/cm^2^ Forward saturation current = 0.14 nA/cm^2^	
[98]	Sensors embedded in soft fabrics	Reflectance oximetry	HR, SpO_2_					Measurement compared graphically with commercial oximeter measurements	
[99]	Wireless oximeter	Reflectance oximetry	HR, RR, SpO_2_, PPT	240 Hz	41mm × 36 mm	<150 mA	ZigBee	SNR of IR = 8 SNR of red =3	
[100]	Micro-machined Pt electrodes	Transmittance oximetry	ECG, HR, SpO_2_ and SBP	200 Hz		<35 mA	ZigBee		
[102]	Ring probe, novel distribution of optical sensors around the phalanx	Transmittance oximetry	HR, SpO_2_		Diameter of the finger			Measurement compared graphically with commercial oximeter measurements	
[103]	Wrist band Sensor	Reflectance oximetry	HR, SpO_2_				CC2500 RF TRX	Ratio of change rates of reflected light intensity in two wavelengths (660 nm and 900 nm)	
[104]	Ring-type pulse oximeters	Reflectance oximetry	HR, RR, SpO_2_, PPT				Bluetooth	Correlation between SpO_2_ values measured by the proposed and commercial oximeter	98.26%
[107]	Analog single-chip pulse oximeter		SpO_2_		2.2 mm × 2.2 mm	4.8 mW		Measurement compared with commercial oximeter measurements	Mean diff. ~−1.2% SD = 1.5%
[108]	Forehead mounted sensor	Reflectance oximetry	HR, SpO_2_				WiFi	Measurement compared with commercial oximeter measurements	
[109]	Electronic Patch with an optical biomedical sensor	Reflectance oximetry	PPG, HR, RR	125 Hz	88 mm × 60 mm (× 5 mm)	I < 33 mA P < 99 mW		PPG is measured using Datex pulse oximeter. SpO_2_ is calculated and plotted against optical ratio for calibration, MSE ~ 2.6%	

**Table 8:** Summary of textile electrodes.

Ref.	Proposition	Electrode Type	Size	Base Material	Conductive Material	Technology	Performance	Contact Resistance
[131]	Direct attach and Interposer electrode	Active electrode	20 × 13 mm^2^ (direct-attach) 11.6 × 11.6 mm^2^ (Interposer)	Nonwoven Evolon fabrics	Conductive ink (CMI 112-15)	Screen printing, stenciling, curing, and encapsulation	*PSDs for sitting and jogging are close to Ag/AgCl electrodes*Durable upto 5 washing cycles	
[136]	Active electrodes on woven textiles	Active electrode	28 mm × 23 mm (skin contact area)	Woven textile composed of cotton, polyester and Lycra fibers	Silver polymer paste (Fabinks TC-C-4001)	Screen and stencil printing	The printed active and Ag/AgCl electrodes had very similar rms levels after filtering	
[138]	2 textile nanofiber web electrodes	Dry electrode	9 mm diameter	PVDF Nanofiber Web	Poly (3,4-ethylene-dioxythiophene) (PEDOT)	Electrospinning-vapor phase polymerization	Tested ECG is 95% similar to Ag/AgCl electrodes	~1000 Ω
				PVDF Nano fiber Web	Silver	Silver mirror reaction	Tested ECG is ~92% similar to Ag/AgCl electrodes	~100 Ω
[139]	Nano copper loaded poly-propylene based textile electrode	Dry electrode	4 cm × 6 cm	Polypropylene nonwoven fabric	Copper nanoparticles on fabric	Multiple dip chemical processes	Max conductivity: 142.8 kΩ·m	
[143]	8 types of electro-thread	Dry fabric electrode	2 × 2 cm^2^, 2 × 5 cm^2^	Polyester 75 denier	Silver thread	Inclusion of one strand or two strands of 50 μm silver thread		32 kΩ at 120 Hz (for 2 Ag strand based 1300TM polyester fabric)
[141]	Several textile-based electrodes	Dry fabric electrode	1.5 cm × 3 cm	PU laminated or dry- coated nylon	Copper coating	Sputtering		5.7 Ω (PU laminated nylon), 10.26 Ω (PU dry-coated nylon).
				Ripstop, Mesh fabric	Cu/Ni coating	Electroless Plating		0.23 Ω/sq (Ripstop), 0.29 Ω/sq (Mesh)
			5 cm × 5 cm	Cotton, Steel/cotton	Stainless Steel Filament Yarn	Embroidering or Knitting	R peak detection accuracy: 58.8% and 64.2%	32.55 Ω/m (linear resistance)
[142]	Knitted fabric electrodes	Dry electrodes	20 mm × 20 mm	Wool and polyester	Silver coated nylon, stainless steel yarn, and silver coated copper	Knitting	FFT response of the multifilament electrodes retains ECG spectralcomponents	
[144]	Embroidered textile electrode	Wet, moisturized by water vapor using the polyester wetting pad.	2 cm × 7 cm	Polyethylene terephthalate yarn of 50 μm diameter	Silver and ultra-thin titanium	Coating by plasma sputtering	Similar signal quality and signal strength after 1 h as after 72 h of use	

**Table 9:** Summary of textile based temperature sensors.

Ref.	Proposition	Type	Fabrication Method	Temperature Range	Sensing Material	Sensitivity	Size	Substrate/Embedding Platform	Performance	Nominal Resistance
[160]	Polymer sensor	Thermistor	Screen printing		Carbon polymer paste			Polyamide foil Kapton	High flexibility, linear characteristic, high thermal resistance change	
[166]	Optical fiber Bragg grating based sensor	Optical	Grinding, polishing	33 °C to 42 °C	Fiber Bragg grating	0.15 nm/°C		Encapsulated with polymer (copolymerization of unsaturated Methyl Ethyl Ketone Peroxide (MEKP) and cobalt naphthenate) filled strip.	Accuracy ~± 0.18 °C	
[165]	Printed sensors on flexible substrate	RTD	Screen printing	20 °C to 80 °C	PTC and NTC resistive pastes	0.025 V/°C at 37 °C	320 mm × 380 mm	Poly Ethylene Naphtalate (PEN)		
[161]	Inkjet printed flexible sensor	Thermistor	Inkjet printing	20 °C to 60 °C	Silver	4.5 Ω/°C at 38.5 °C	2.85 cm × 2.26 cm	Polyimide substrate (Kapton HN)	Good linearity (coefficient of linearity ~ 0.9998) Hysteresis less than 5%	2.032 kΩ at 38.5 °C
[158]	Arrays of single sensors on a flexible substrate	RTD	Electron beam evaporation followed by photolitho-graphy	25°C to 90°C	Meander shaped structures of platinum	1.52 Ω/°C	67.5 mm × 67.5mm	Kapton E foils, Integrated into textile using weaving	The sensors damage at strong bending of around 11% due to cracking of the sensing lines	
[164]	Sensors on paper substrate	RTD	Inkjet printing	−20 °C to 60 °C	Silver nano-particles		16 mm × 16 mm	Nano-porous oxide film coated paper	Good linearity with a TCR of 0.0011/°C, with perylene coating linearity, is 0.9999, resistivity 30 µΩ·cm	740 Ω with perylene coating
[159]	Embroidered sensors	RTD	Embroidery	20 °C to 100 °C	Conductive yarn made of austenitic Cr-Ni stainless steel wires	2.68 Ω/°C	90 mm × 90 mm	Embroidered on a textile substrate	Good resistance against washing cycles	
[162]	Printed wearable sensor	RTD	Shadow mask printing	22 °C to 50 °C	Mixer of carbon nanotube and PEDOT:PSS	0.6 %/°C		SiO_2_-coated Kapton	Good stability, highly sensitive	
[163]	Ultrasensitive wearable sensor	RTD	PECVD and polymer-assisted transfer method	35°C to 45°C	Grapheme nanowalls		20 mm × 10 mm	Polydimethylsiloxane (PDMS)	TCR = 0.214/°C, response time 1.6 s and recovery time 8.52 s	706.2 Ω at 25 °C
[153]	Flexible wireless sensors	RTD with integrated passive RFID antenna		35 °C to 42 °C	Ni microparticle- filled binary polymer (polyethylene (PE) and polyethylene oxide (PEO)) composites	0.1 to 0.3 V/°C			Accuracy ~± 2.7 °C	
[157]	Temperature sensing fabric	RTD	Metal wire inlaid in the middle of a rib knitted structure	20 °C to 50 °C	Platinum wire, Diameter < 25 mm		8 cm × 8 cm	Polyester fabric	Coefficient linearity in the range of 0.99–0.999	3 Ω to 130 Ω

**Table 10:** Summary of textile-based strain sensors.

Ref.	Proposition	Sensing Mechanism	Structure/Base	Sensing Material	Gauge Factor	Stable Strain Range	Demonstrated/Potential Applications
[171]	Textile-structured flexible strain sensor	Contact resistance of fiber/yarn/fabric	Single warp fabric	Carbon fiber	10–200 depending on fiber length	Max 200%	Wearable strain sensor
[172]	Textile-based strain sensor	Contact resistance of conductive fiber loops	Fabric with elastomeric yarns	Silver coated polymeric yarn made loops	0.75	40%	Wearable strain sensor
[168]	Stretchable and Sensitive Strain Sensor	Piezoresistive	PDMS	Ag nano-walls thin film	2 to 14	70%	Finger movements
[170]	Textile-based strain sensor for monitoring the elbow and knee movements	Piezoresistive	Elastic yarns made from Lycra fiber wrapped with two polyester yarns.	Carbon particles coated polyamide fiber twisted with polyester yarn	~0.3	30%	Flexion angle of elbow and knee movements
[169]	Stretchable strain sensor based on a metal nanoparticle thin film for human motion detection	Piezoresistive	PDMS	Silver nanoparticle	2.5	20%	Finger movements
[175]	Knee’s kinematic monitoring using single optical FBG sensor	Fiber Bragg grating	Optical Fiber	Polymer encapsulated FBG sensor	~0.8	0.04%	Knee, finger movements, HR, RR
[176]	Force sensors based on light pipes in the form of multimode optical fibers made of copolymers.	Loss of light due to deflection of the fiber with force	Multimodal optical fiber	Copolymers containing silicon and polyurethane			Force sensing
[167]	Textile-based MEMS accelerometer	Piezoresistive	Cotton fiber	Silver nanoparticles	7.796 ± 2.835		Motion sensing
[174]	All-polymeric knitted textile strain sensor	Piezoresistive	Commercial Spandex yarn	PU/PEDOT:PSS fibers	0.2 to 1	160%	Knee bending movements

**Table 11:** Key features of currently available wireless technologies.

Wireless Technology	Frequency Band	Range	Data Rate	Power Consumption	Maximum Number of Nodes Supported	Supported Network Topologies	Security	Modulation	Reference
RFID	13.56 MHz 860–960 MHz	0-3 m	640 kbps	200 mW	1 at a time	P2P (passive)	N/A	ASK, PSK, FSK	[73,74,196]
Bluetooth	2.4–2.5 GHz	1–100 m	1–3 Mbps	2.5–100 mW	1 master + 7 slave	P2P, star	56–128 bit key	GFSK	[29,30,45,59,66,87,90,91,92,93,104]
BLE	2.4–2.5 GHz	1–100 m	1 Mbps	10 mW	1 master + 7 slave	P2P, star	128-bit AES	GFSK	[28,88]
ZigBee	2.4–2.5 GHz	10–100 m	250 kbps	35 mW	65,533	P2P, star, tree and mesh	128-bit AES	OQPSK, BPSK	[70,99,100]
WiFi	2.4–2.5 GHz	150–200 m	54 Mbps	1 W	255	P2P, star	WEP, WPA, WPA2	BPSK, QPSK, QAM	[108]
UWB	3.1–10.6 GHz	3–10 m	53–480 Mbps	250 mW	1 master + 7 slave	P2P, star		BPPM, FSK	
ANT	2.4–2.5 GHz	30 m	20–60 kbps	0.01–1 mW	65,533 in one channel	P2P, star, tree and mesh	64-bit key	GFSK	[10,19,188]
MICS	402–405 MHz	2 m	200–800 kbps	25 µW		P2P, star		FSK	
IrDA	38 kHz	10 cm	1 Gbps		1 at a time	P2P			
NFC	13.56 MHz	5 cm	424 kbps	15 mW	1 at a time	P2P	AES	ASK	
